# A New Varunid Subfamily (Decapoda, Brachyura, Grapsoidea, Varunidae) for Crabs From European and West African Waters, With the Description of Two New Genera and Two New Species

**DOI:** 10.1002/ece3.71712

**Published:** 2025-07-22

**Authors:** Isabel Muñoz, J. Enrique García‐Raso, Jose A. Cuesta

**Affiliations:** ^1^ Centro Oceanográfico de Cádiz (Instituto Español de Oceanografía) Consejo Superior de Investigaciones Científicas Cádiz Spain; ^2^ Departamento Biología Animal, Facultad de Ciencias Universidad de Málaga Málaga Spain; ^3^ Instituto de Ciencias Marinas de Andalucía (ICMAN‐CSIC) Avda. República Saharaui 2 Puerto Real (Cádiz) Spain

**Keywords:** Asthenognathinae, *Asthenognathus atlanticus*, biogeographic distribution, eastern Atlantic, speciation process

## Abstract

The subfamily Asthenognathinae is currently composed of a single genus, *Asthenognathus* Stimpson, 1858, with three species, *A. inaequipes* Stimpson, 1858, *A. hexagonus* Rathbun, 1909 (both from the Indo‐West Pacific), and *A. atlanticus* Monod, 1933 (European and West Tropical African waters). All *Asthenognathus* species are difficult to collect due to their small size and symbiotic lifestyle with fossorial organisms. The examination of specimens of *A. atlanticus* from European and West African waters deposited in scientific collections shows significant morphological and molecular differences with the Indo‐West Pacific species, which makes the creation of a new subfamily (Schubartinae n. subf.) and two new genera necessary. The identity of *A. atlanticus,* the only species found along the East Atlantic and Mediterranean waters, is clarified, and a new genus, *Dudekemus* n. gen., is established for this species, *Dudekemus atlanticus* (Monod, 1933) n. gen., n. comb. This study also describes another new genus, *Schubartus* n. gen., based on morphological differences and genetic data. These two new genera can be distinguished from *Asthenognathus* by the carapace proportions and differences in the structures of the male pleonal somites, pereiopods and endostome. In addition, two new species distributed in Guinea‐Bissau and Mauritania, *Schubartus mauritanicus* n. gen., sp. nov., and *S. ngankeeae* n. gen., n. sp., are described. A key for species of the subfamilies Asthenognathinae and Schubartinae n.subf. is provided.

## Graphical abstract

Morphological and DNA sequencing studies of Varunidae specimens, previously considered to belong to the genus Asthenognathus, from eastern Atlantic waters have allowed the description of two new genera and two new species. From a molecular point of view, it is shown that they belong to a new subfamily, distinct from Asthenognathinae and that there are still unresolved phylogenetic relationships within the Varunidae family, which will lead to the reorganisation and creation of new taxa. The distribution of these species in Atlantic ecoregions and diversity hotspots is discussed.

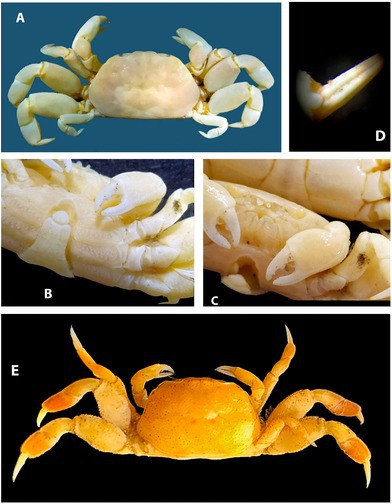

## 1 Introduction

The family Varunidae H. Milne Edwards, 1853, in its current composition, is the result of studies considering adult and larval morphology, as well as molecular data (Cuesta et al. 2000; Schubart et al. 2000, 2001, 2006; Ng 2006; Palacios‐Theil et al. 2009; Poore and Ahyong 2023). According to Poore and Ahyong (2023) (following Davie and Ng 2007; Ng et al. 2008; Davie et al. 2015a; Palacios‐Theil et al. 2009), six subfamilies are included in Varunidae: Asthenognathinae Stimpson, 1858; Cyclograpsinae H. Milne Edwards, 1853; Gaeticine Davie & Ng, 2007; Pinnotherelinae Alcock, 1900; Thalassograpsinae Davie & Ng, 2007; and Varuninae H. Milne Edwards, 1853.

There are two genera, *Hemiplax* Heller, 1865, and *Tritodynamia* Ortmann, 1894, which Ng et al. (2008) and Poore and Ahyong (2023) included within Macrophthalmidae Dana, 1851, but Tsang et al. (2022), in their molecular phylogeny of Thoracotremata, place these two genera in Varunidae, clearly separate from Macrophthalmidae. *Hemiplax* is considered varunid, following McLay et al. (2010) and Kitaura et al. (2002, 2010); also, according to Ng (personal communication), the positions of their gonopores are clearly those of varunids, not macrophthalmids, although their taxonomic position is not clear and perhaps they should be in their own subfamily.

Sakai et al. (2006) and Guinot et al. (2018) recognise the subfamily Heliceinae Sakai, Türkay & Yang, 2006, within Varunidae, for some genera included in Cyclograpsinae, but this subfamily is not yet included in WoRMS and DecaNet, nor in Poore and Ahyong (2023).

Despite systematic studies on Varunidae and closely related families, there are still a number of unresolved questions to clarify (Cuesta et al. 2005; Ng 2006; Davie and Ng 2007; Ng et al. 2008; Tsang et al. 2022). Furthermore, it is unclear whether this family is a monophyletic group (Guinot et al. 2018) and needs a thorough revision.

Asthenognathinae currently comprises a single genus, *Asthenognathus* Stimpson 1858, although previously four genera belonged to this subfamily: *Asthenognathus*, *Tritodynamia*, *Aphanodactylus* Tesch, 1918, and *Voeltzkowia* Lenz, 1905 (see Ng et al. 2008). The taxonomic position of the last three genera has been changing in recent years: *Aphanodactylus* (presently accepted as *Selwynia* Borradaile, 1903) was placed in Aphanodactylidae Ahyong & Ng, 2009; *Tritodynamia* is in Macrophthalmidae in the subfamily Tritodynamiinae Števčić, 2005 (see Števčić 2005; Ng and Ho 2023; Poore and Ahyong 2023) and *Voeltzkowia* is presently accepted as *Gandoa* Kammerer, 2006, also placed in Aphanodactylidae (Ahyong and Ng 2009; Ng and Rahayu 2016).

The genus *Asthenognathus* was previously considered part of Pinnotheroidea De Haan, 1833 (Dai and Yang 1991), but Ng et al. (2008), following the suggestion by Cuesta et al. (2005) and after a morphological re‐examination, transferred it to Varunidae in its own subfamily, and later, Palacios‐Theil et al. (2009), based on molecular evidence, confirmed this placement. Initially, this genus included four extant species: three from the Indo‐West Pacific: *Asthenognathus inaequipes* Stimpson, 1858 (type species); *A. hexagonus* Rathbun, 1909 (in Decanet and previous works, this species is called *Asthenognathus hexagonum*; but, as the gender is masculine, the species should be called *A. hexagonus* (Ng PKL, personal communication)), and *Asthenognathus gallardoi* Serène & Soh, 1976, and a fourth, *A. atlanticus* Monod, 1933, from the Eastern Atlantic and Western Mediterranean (Ng et al. 2008; Naruse and Clark 2009; DecaNet 2024). However, *A. gallardoi* was considered a new genus, *Gopkittisak* Naruse and Clark 2009 by Naruse and Clark (2009), and was transferred to the subfamily Gaeticinae (see also Lasley Jr et al. (2024)).

All *Asthenognathus* species are difficult to collect due to their small size and symbiotic lifestyle. In general, they inhabit mud bottoms (Glémarec and Hily 1979; Jourde et al. 2012) and are associated with a benthic fauna where echinoderms, annelids, and small crustaceans predominate (Monod 1933; Glémarec and Hily 1979; Jourde et al. 2012; Pezy and Dauvin 2016; Faasse et al. 2021). Recently, *A. inaequipes* was collected for the first time in a burrow of the holothurian *Protankyra bidentate* (Woodward & Barrett, 1858) (see Lee et al. 2010).

In European and West Tropical African waters, only *A. atlanticus* has been recorded, with its known distribution ranging from the southern North Sea (Bruine Bank, Netherlands), Normandy, the English Channel, and North‐West France to Spain, West Africa (Morocco to Angola) and the Western Mediterranean Sea (Monod 1956; Manning and Holthuis 1981; d'Udekem d'Acoz 1999; Faasse et al. 2021).

In the present study, specimens of *A. atlanticus* collected in Mauritania, Guinea‐Bissau, and Ivory Coast have been studied and compared with representatives from other localities in European and African waters, as well as specimens of *A. inaequipes* and *A. hexagonus* from different collections. Molecular analyses (using 16S and COI genetic markers) were carried out, and comparison was extended to other varunids to obtain a better understanding of their intrafamilial relationships.

## 2 Materials and Methods

Specimens included in this study come from different sources, and all of them are deposited in various Natural History Collections, such as the Naturalis Biodiversity Center (NBC, Leiden, Netherlands) (Bakker et al. 2024), the Muséum national d'Histoire naturelle (MNHN, Paris, France) (Chagnoux 2024), the Natural History Museum of Denmark (NHMD, Copenhagen, Denmark) (Eibye‐Jacobsen et al. 2024), the Senckenberg Museum Frankfurt (SMF Frankfurt/Main, Germany) (Senckenberg 2024), and the Marine Crustacean Collection of Instituto Español de Oceanografía, Cádiz Oceanographic Center (CRUST_IEOCD, Cádiz, Spain), including the Dr. García‐Raso personal collection donated to CRUST_IEOCD (Muñoz 2024).

### 2.1 Morphological Analysis

The terminology used in the descriptions and comparisons follows Davie et al. (2015b). Measurements are provided in millimetres and are reported as CL, maximum carapace length along the dorsal midline from the base of the rostral sinus to the posterior margin of the carapace, and CW, maximum carapace width taken at its widest point. The length (L) of the segments of the appendages has been taken by their latero‐longitudinal midline and their height (H) by the median part.

Other anatomical abbreviations used in the text and figures are: indet: indeterminate; juv: juvenile; ovig: ovigerous; P1–P5: first to fifth pereiopods (P1, chelipeds; P2–P5, ambulatory legs 1–4); the thoracic somites are numbered from s1 to s8; pleonal somites are numbered from so1 to so6; G1–G2: first and second gonopods; T: telson. Others: Stn: station; ♀: female; ♂: male.

### 2.2 Molecular Analysis

To prevent damage to key structures needed for morphological identification, only small portions of muscle tissue from a single ambulatory leg were used for DNA extraction in males and non‐ovigerous females, while eggs were used for ovigerous females.

Extraction protocols follow that from Estoup et al. (1996) for some samples, while for older samples and those from museums and collections, a modified protocol using the Qiagen DNeasy Blood & Tissue Kit was adopted and carried out at the Instituto de Ciencias Marinas de Andalucía (ICMAN‐CSIC). Partial sequences of the mitochondrial 16S rRNA and cytochrome c oxidase subunit I (COI) genes were amplified. The cycling conditions of the polymerase chain reaction (PCR) using the Surf Hot Taq (Stab Vida company) were initially 15 min at 95°C and 35 cycles of (1) 30 s at 95°C, (2) 30 s (16S) or 45 s (COI) at 45°C–54°C (16S—depending on primer) or 43°C–51°C (COI—depending on primer), and (3) 30s (16S and short sequences of COI) or 45 s (COI) at 72°C, and finally 5 min at 72°C. The primers used, including newly designed ones, are listed in Table 1. Some PCRs were carried out using a TouchDown protocol where the annealing temperature is selected 10°C higher than the melting temperature of the primer, and after each cycle (during the first 10 cycles), the temperature is decreased by 1°C (see Green and Sambrook 2018).

**TABLE 1:** List of sequenced genes including primers used for each gene, pair combinations, length of the sequences obtained (bp), and references.

Genes	Primers (forward and reverse)	Pair	(bp)	References
16S	16SL29 (5′‐YGC CTG TTT ATC AAA AAC AT‐3′)	1472	540	Schubart et al. (2000)
	16 L12 (5′‐TGA CCG TGC AAA GGT AGG ATA A‐3′)	1472	450	Schubart et al. (1998)
	16L12c (5′‐TGA CYG TGC AAA GGT AGS ATA A‐3′)	1472	450	This study, modified from 16 L12 primer (Schubart et al. 1998)
	MiDeca‐Fb (5′‐RGA CGA TAA GAC CCT RTA AA‐3′)	1472	300	This study, modified from MiDeca‐F/R primers (Komai et al. 2019)
	1472 (5′‐AGA TAG AAA CCA ACC TGG‐3′)	16L2	570	Crandall and Fitzpatrick (1996)
	MiDeca‐Rb (5′‐ACG CTG TTA TCC CTK KAG T‐3′)	16L12c	300	This study, modified from MiDeca‐F/R primer (Komai et al. 2019)
COI	COL6b (5′‐ACA AAT CAT AAA GAT ATY GG‐3′)	COH6	658	Schubart and Huber (2006)
	COH6 (5′‐TAD ACT TCD GGR TGD CCA AAR AAY CA‐3′)			Schubart and Huber (2006)
	LCO1490 (5′‐GGTCAACAAATCATAAAGATATTG‐3′)	HCO2198	658	Folmer et al. (1994)
	HCO2198 (5′‐TAAACTTCAGGGTGACCAAAAAATCA‐3′)			Folmer et al. (1994)
	LCO1490‐JJ (5′‐CHA CWA AYC ATA AAG ATA TYG G‐3′)	HCOI‐2198JJ	658	Astrin and Stüben (2008)
	HCO2198‐JJ (5′‐AWA CTT CVG GRT GVC CAA ARA ATC A‐3′)			Astrin and Stüben (2008)
	mlCOIintF‐VARU (5′‐CAG GRT GRA CHG THT AYC CYC C‐3′)	COH6	320	This study, modified from mlCOIintF‐XT primer (Wangensteen et al. 2018).
	mlCOIintR‐VARU (5′‐GGR GGR TAD ACD GTY CAR CCT G‐3′)	COL6b	333	This study, modified from mlCOIintF‐XT primer (Wangensteen et al. 2018)

PCR products were sent to Stab Vida laboratories to be purified and then bidirectionally sequenced. The resulting forward and reverse reads were assembled, edited and consensus sequences extracted using GENEIOUS v.10.0.9 (Kearse et al. 2012). MAFFT v.7.402 server (Katoh and Standley 2013) was employed to align the sequences using the L‐INS‐i iterative refinement algorithm via the CIPRES Portal Science Gateway (Miller et al. 2010). The final DNA sequences obtained were compared with sequences retrieved from the GenBank database. New sequences have been deposited in GenBank under the accession numbers PQ247075–PQ247087 (16S) and PQ247033–PQ247043 (COI).

Bayesian inference (BI) and maximum likelihood (ML) analyses were conducted for the concatenated dataset with the two genes, including the new sequences obtained in the present study, as well as sequences of other related species downloaded from GenBank (see Table 2). In the analysis were included representatives from all varunid subfamilies as well as other grapsoidean families, although it was not possible to find available sequences for all varunid subfamilies for COI. Two species of Ocypodidae were used as outgroups, considering that they are Thoracotremata but of a different superfamily.

**TABLE 2:** List of specimens used in the molecular analysis of this study, including specimen code of the institution where the DNA voucher is deposited, and accession codes of the sequences deposited in the GenBank database.

Specimen id.	Specimen code	16S	COI
*Afruca tangeri*	NCHU:ZOOL:13585/14,911	AB813666	LC150399
*Armases cinereum*	ULLZ 4392/UF:68451b	AJ784010	OQ396649
*Asthenognathus inaequipes*	(—)	NC_063603^a^	NC_063603^a^
*Brachynotus sexdentatus*	(—)	KU246045	(—)
*Cardisoma carnifex*	NTOU‐LMT‐BRA0037/NCHUZOOL 13599	ON379426	OQ197467
*Chasmagnathus convexus*	RL267‐10/NCHUZOOL13101	ON379443	AB334556
*Dudekemus atlanticus* n. comb.	CRUST‐JEGR/3921	**PQ248975**	**PQ247033**
*Dudekemus atlanticus* n. comb.	IEOCD‐AR13/845	**PQ248976**	**PQ247034**
*Dudekemus atlanticus* n. comb.	CRUST‐ICMAN/3923	**PQ248977**	**PQ247035**
*Dudekemus atlanticus* n. comb.	CRUST‐JEGR/3928	**PQ248978**	(—)
*Dudekemus atlanticus* n. comb.	RNMH.CRUS.D57968	**PQ248979**	**PQ247036**
*Dudekemus atlanticus* n. comb.	CRUST‐JEGR/3927	**PQ248980**	**PQ247037**
*Dudekemus atlanticus* n. comb.	(—)	(—)	ILGEA283‐23
*Gaetice americanus*	ULLZ 4106	AJ250643	(—)
*Gecarcinus quadratus*	MSLKHC‐BRA243	ON379427	(—)
*Geograpsus lividus*	CCDB1518	KU313179	(—)
*Glyptograpsus jamaicensis*	SMF25987	AJ308420	(—)
*Hemigrapsus nudus*	KH_1302/BIOUG14663‐E02	MW363097	MF745735
*Ocypode africana*	SMF9823/(—)	LC150354	MH615041
*Pachygrapsus gracilis*	R382‐4/CCDB < BRA >:811	KM510115	MF490106
*Percnon gibbesi*	ULLZ13479	MK971658	MN184217
*Percnon guinotae*	SMF24946/USNM:1467486	FN539015	MZ559694
*Pinnotherelia laevigata*	ULLZ14798	KU679733	(—)
*Plagusia dentipes*	SMF24559/CASMKAK‐07	AJ308421	KY284645
*Plagusia speciosa*	MNHN:B‐30681	FN539008	(—)
*Platychirograpsus spectabilis*	SMF24567	AJ250645	(—)
*Ptychognathus barbatus*	MSLKHC‐BR138/MNHN‐IU‐2019‐210	ON379449	OR864745
*Schubartus mauritanicus* n. gen., n. sp.	RNMH D‐40008‐1	**PQ248981**	**PQ247038**
*Schubartus mauritanicus* n. gen., n. sp.	RNMH D‐40008‐2	**PQ248982**	**PQ247039**
*Schubartus ngankeeae* n. gen., n. sp.	RNMH.CRUS.D40008‐3	**PQ248983**	**PQ247040**
*Schubartus ngankeeae* n. gen., n. sp.	RNMH.CRUS.D39701	**PQ248984**	**PQ247041**
*Schubartus ngankeeae* n. gen., n. sp.	RNMH.CRUS.D40005	**PQ248985**	**PQ247042**
*Schubartus ngankeeae* n. gen., n. sp.	IEOCD‐CCLME12/1639‐1	**PQ248986**	(—)
*Schubartus ngankeeae* n. gen., n. sp.	IEOCD‐CCLME12/1639‐2	**PQ248987**	**PQ247043**
*Sesarma rectum*	UF8827b	OQ401448	OQ396667
*Thalassograpsus harpax*	OMNH Ar 7681	AB440192	(—)
*Varuna litterata*	MSLKHC‐BR192/(—)	ON379451	AB334556

*Note:* Sequences obtained in the present work are in bold. (—) no sequence or data available.

^a^ Sequence extracted from the complete mitogenome.

The best‐fit models of evolution were determined for 16S and COI genes using the Akaike information criterion (AIC) (Akaike 1998) implemented in jModelTest 2.1.10 (Darriba et al. 2012). Bayesian inference analyses were performed for the concatenated datasets using the software package MrBayes v.3.2.6 (Ronquist et al. 2012) for 7 million generations, with two independent runs with four MCMC chains, a sampling frequency of 1000 generations and a ‘burn‐in’ of 25%. Nodal support was estimated as posterior probabilities (PP), with values ≥ 90% taken as significant (Huelsenbeck and Rannala 2004). ML analyses were performed using the software RAxML 8.2.10 (Stamatakis 2006). Node support was assessed with non‐parametric bootstrapping (BS) with 1000 replicates, random starting trees and parameters estimated from each dataset under the model selected for the original dataset. Values ≥ 70% were considered statistically significant. The trees obtained were visualised in FigTree v.1.4.2 (Rambaut 2012).

## 3 Results

### 3.1 Molecular Results (Figure 1)

**FIGURE 1:**
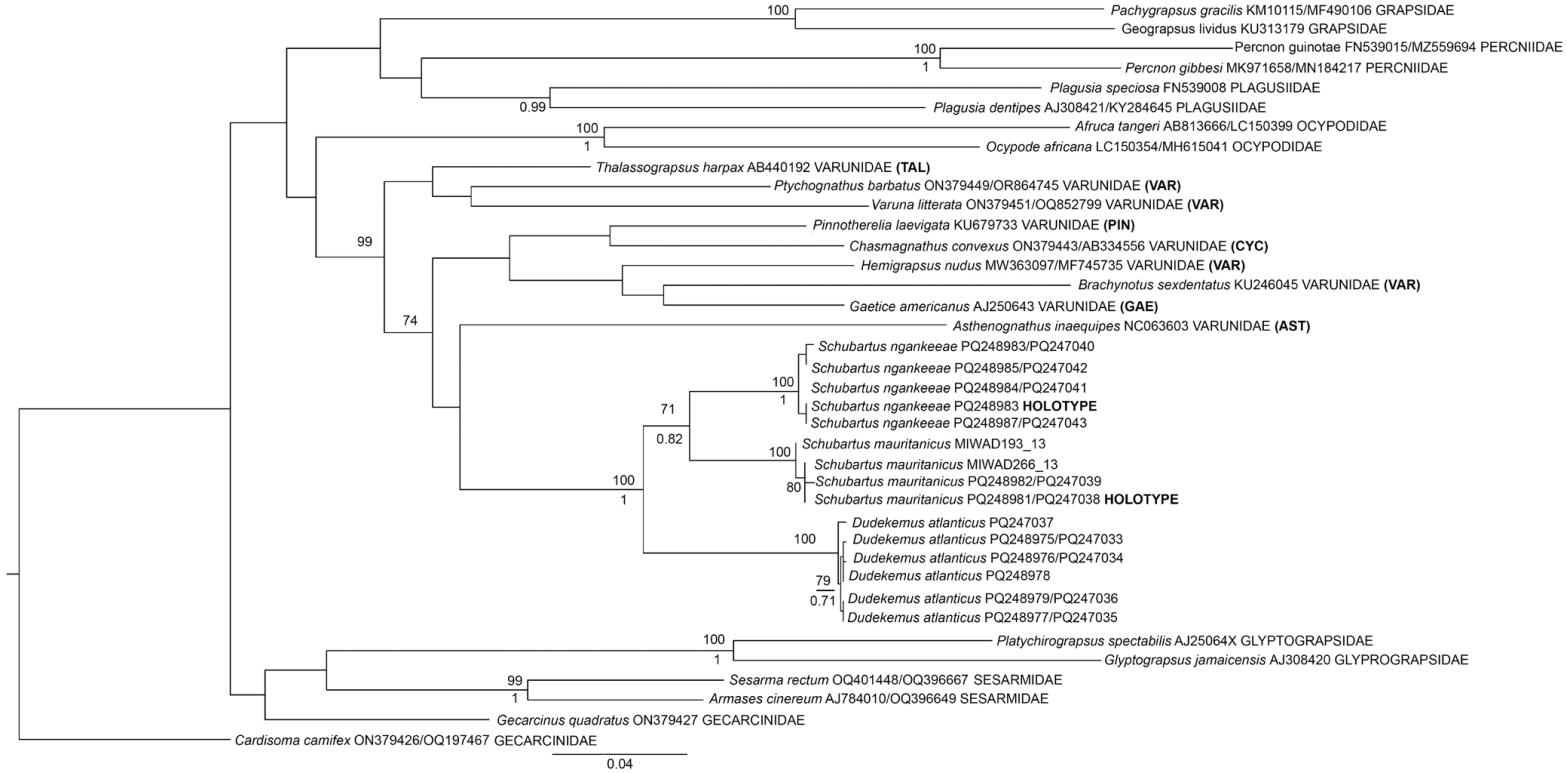
Molecular phylogeny based on Bayesian inference (BI) analysis of a concatenated alignment of mitochondrial 16S rRNA and cytochrome c oxidase subunit I (COI) sequences of species in the genera *Dudekemus* n. gen., *Schubartus* n. gen., and representatives of all Varunidae subfamilies, and representatives of several Grapsoidea families. Significant support values are given as maximum likelihood (ML) bootstrasp percentages (BS, above branch) and BI posterior probabilities (PP, below branch). *Afruca tangeri* and *Ocypode africana* are used as outgroups. GenBank accession codes (16S and/or COI) are indicated for each species. Abbreviations after Varunidae refer to subfamily: AST, Asthenognathinae; CYC, Cyclograpsinae; GAE, Gaeticinae; PIN, Pinnotherelinae; TAL, Thalassograpsinae; and VAR, Varuninae.

The complete concatenated alignment consisted of 1248 positions, 590 (16S) and 658 (COI). The evolutionary model selected for each gene was the HKY + I + G for 16S and GTR + Ι + Γ for COI. All sequences included in the analysis are listed in Table 2.

The results of the analysis show a clearly separate and well‐supported clade for the two new genera and two new species (Figure 1). This clade is clearly distanced from the representative of Asthenognathinae and other varunid genera (Figure 1). The analysis shows that intrafamilial relationships in Varunidae are not well solved, and at this point the new genera cannot be included in any current varunid subfamilies, and are clearly not related to Asthenognathinae; therefore, we propose the creation of a new subfamily, Schubartinae n. subf.

### 3.2 Morphological Results

#### 3.2.1 Systematic

**Family Varunidae** H. Milne Edwards, 1853.

**Subfamily Schubartinae** n. subf.

(urn:lsid:zoobank.org:act:0C334E00‐C9E3‐40C3‐9B2D‐F67BA6C6F0EA)

*Type Genus*: *Schubartus* n. gen., by present designation.

*Diagnosis*: Carapace broadly trapeziform, wider than long, maximum carapace width towards the posterior part of the carapace (third quarter), regions poorly defined with an evident cervical groove, orbital angle rounded and anterolateral tooth indistinct, but with sinuosity. Slightly bilobed front. Orbit close or open laterally in long transverse depression; infraorbital ridge‐groove running laterally upwards. Third maxillipeds forming a gape when closed; ischium with setae in the inner margin with visible longitudinal sulcus, also on the merus; merus as long as wide or slightly longer; palp with carpus > dactylus > propodus. Epistome short, narrow, triangular. Chelipeds are robust in male adults. P2–P5 long, ventrally finely granulated; P3 and P4 longer; P5 reduced in size. Male so1–so6 movable. Adult male pleon with so6 tapering distally to meet the proximal part of the telson, which is narrower. G1 thick, curved outwards, distal somewhat narrower, surrounded by a crown of setae. G2 slender, small. Vulvae not exposed externally, fully concealed by the folded pleon, large, with operculum, with thick margins, occupying the upper third and distant from the midline of s6.

*Comparative Material*: *Asthenognathus hexagonus* (holotype, ovigerous ♀, CW × CL: 7.7 × 5.7 mm), ZMUC_CRU_6704, 23‐01‐1900, 3.2 km N of Ko Kong (Gulf of Thailand), 14.6 m, from the Natural History Museum Denmark (NHMD, Copenhagen, Denmark). *Asthenognathus inaequipes*, 1 ♂ 8.5 × 5.9 mm, Crustacea_SMF_30931, Shimabara, Mizuho (Japan), 19‐07‐1989, from the Senckenberg Museum (Frankfurt/Main, Germany).

*Remarks*: The molecular study shows that the specimens of the only known Atlantic species of *Asthenognathus* belong to three species (two new), and they should be assigned to two new genera since the molecular distances with the type species of the genus *Asthenognathus* are very large (Figure 1). In addition, there are morphological differences with the other subfamilies of Varunidae:

(1) Schubartinae differ from Asthenognathinae because the carapace is always trapezoidal, with maximum width towards the posterior part (3/4), and the length of the frontal region (anterior part) is less than half the maximum width of the carapace. (2) Varuninae has a carapace that is subquadrate or oval, with the maximum width usually at or close to the exorbital width, while in Schubartinae, it is trapezoidal and much larger, with maximum width at the posterior part of the carapace. (3) Gaeticinae presents: (a) a narrow gape between the third maxillipeds when closed (wider in Schubartinae), (b) a palp markedly elongated, armed with a very long brush of setae (extending into a medial sternal groove) and a strongly inwardly oblique ischiomeral articulation, (c) a sternal plastron with a relatively deep longitudinal sulcus anterior to the pleon, into which the setal brush of third maxillipeds is folded for protection, (d) a male pleon with segments 3 to 6 functionally fused, with sutures sometimes visible (Davie and Ng 2007; Poore and Ahyong 2023), although Davie et al. (2015b) for Varunidae quoted: a male pleon with 6 free somites plus a telson. (4) Thalassograpsinae present (a) a short distinct lateral sulcus in the frontal margin, just posterior to the lateral frontal margin; (b) the third maxilliped without a gape when closed, and the merus and ischium lack a visible longitudinal sulcus (visible in other subfamilies); (c) walking legs with a subapical spine on the posterodistal border of the propodus; (d) a male with abdominal segments 5 and 6 fused, but with the suture visible (Davie and Ng 2007). (5) Cyclograpsinae differs because it shows (a) an oblique setose crest across the merus and distolateral corner of the ischium of maxilliped 3 (absent in others subfamilies); (b) a complex reticulation of setae on the pterygostomian region of the carapace and a deep vertical groove parallel to the buccal cavity. (6) Pinnotherilinae (as in Gaeticinae) has a long setal brush in maxilliped 3 palp, absent in Schubartinae; and male pleonal somites so5 and so6 are fused.

Schubartinae shares a carapace trapezoid with some Asthenognathinae (although it is more hexagonal in the latter), while other subfamilies are subquadrate or oval; third maxillipeds with a gape when closed are also present in Asthenognathinae, Varuninae, Cyclograpsinae, Pinnotherelinae (narrow) and Gaeticinae (narrow), but not in Thalassograpsinae; a visible longitudinal sulcus on the ischium of the third maxilliped exists in all subfamilies, except Thalassograpsinae; and male pleonal somites so1 to so6 are movable, also present in Asthenognathinae, Cyclograpsinae and Varuninae (not in other subfamilies).

**FIGURE 2:**
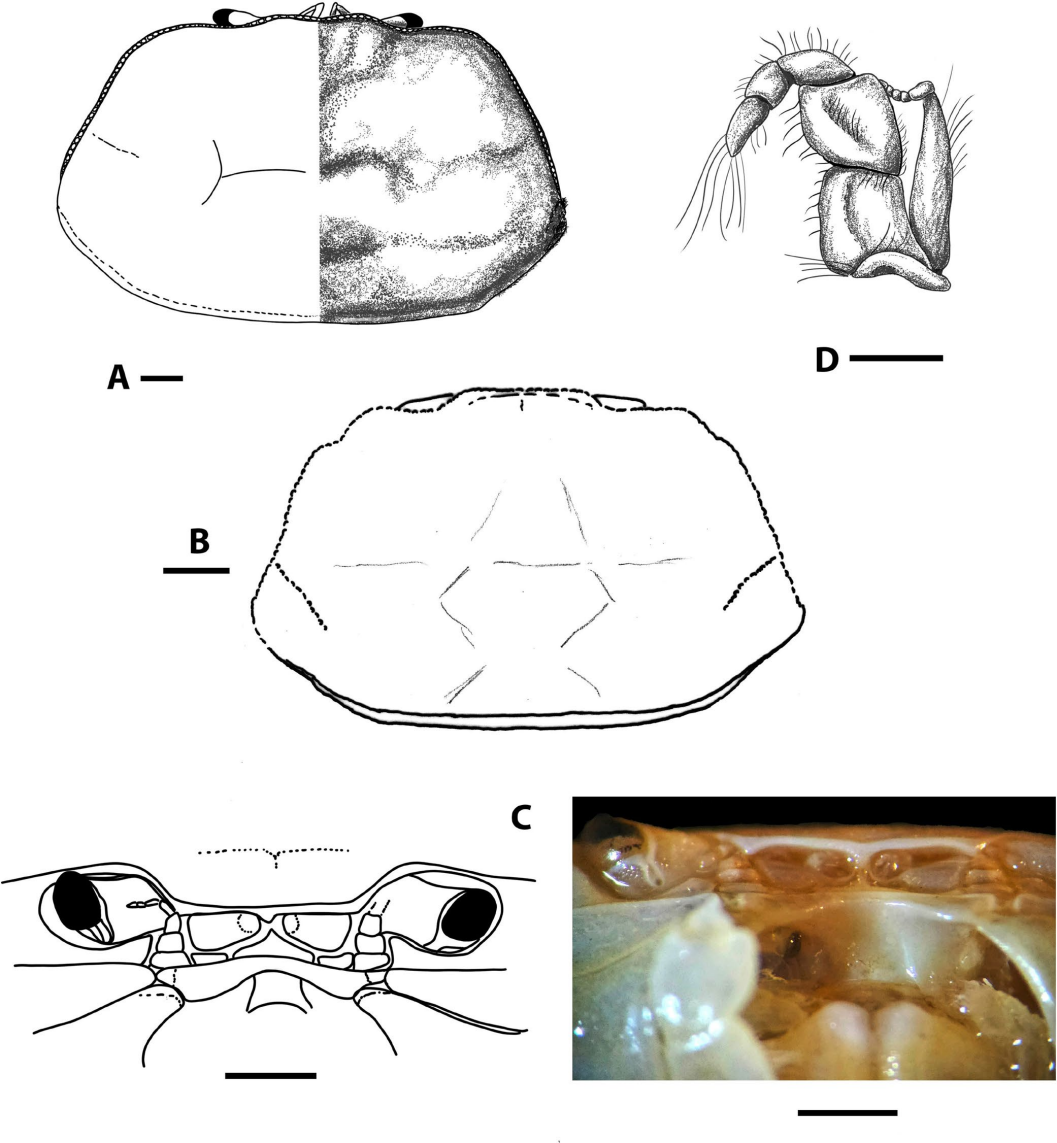
*Dudekemus atlanticus* n. gen., n. comb. (A, B) Carapace, dorsal view; (C) Anterior region, buccal frame (frontal view) and endostome (figure and photo). No setae. (A, D) Female (CRUST_JEGR/3916) from Málaga, 14.1 mm CW; (B) Male (RMNH.CRUS.D.57968), from Bruine Bank, 7.52 mm CW; (C) Female (CRUST_JEGR/3917) from Málaga, 14.5 mm CW. Scales 1 mm.

**FIGURE 3:**
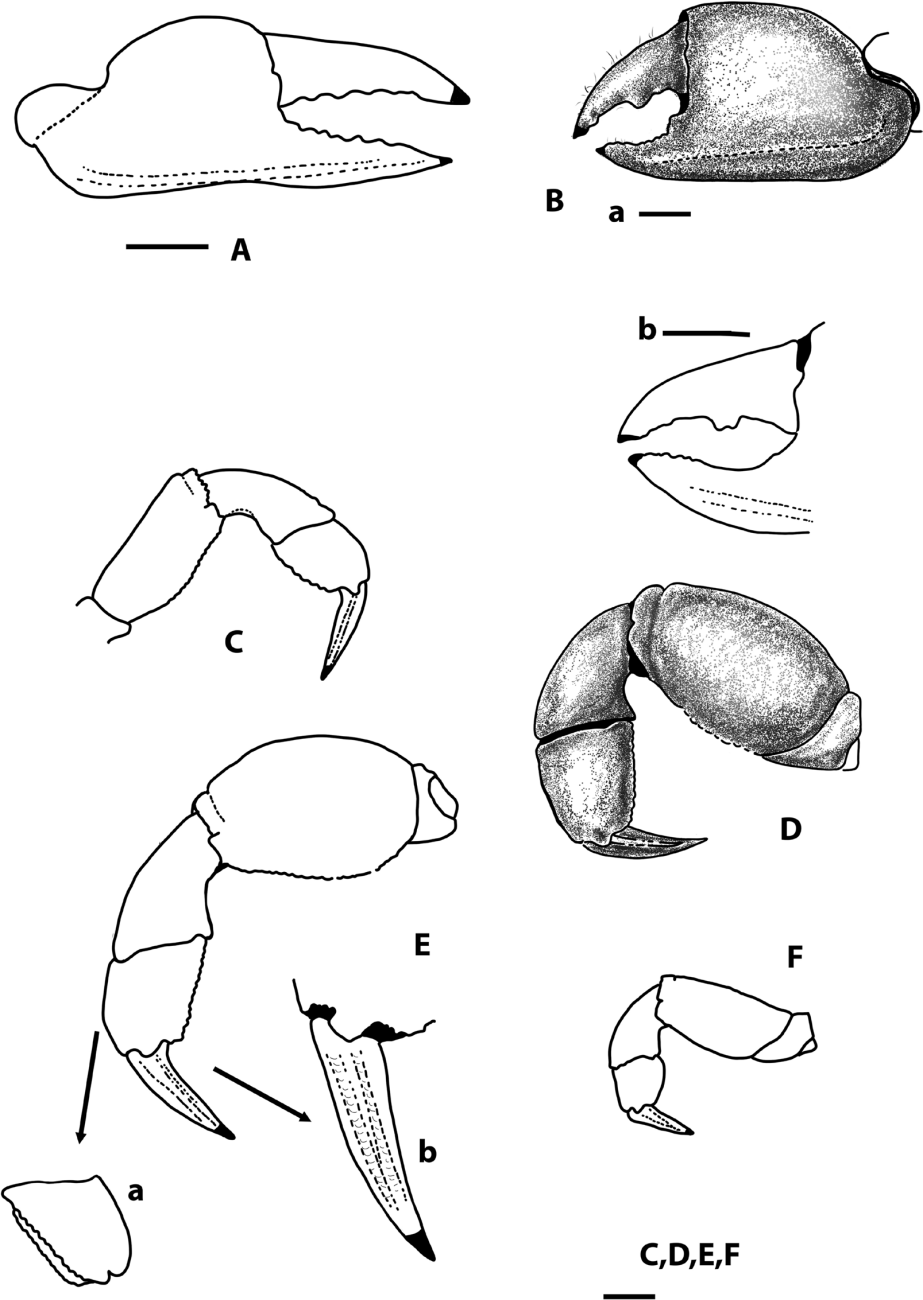
*Dudekemus atlanticus* n. gen., n. comb. (A) Right cheliped, female, outer view; (B) Left cheliped, male, outer view (a) and detail of the fingers (b); (C) P2 right, female, inner view; (D) P3 left, female, outer view; E: P4 left, female, outer view, with inner view of the carpus (a) and detail of the dactylus (b); F: P5 left, female, outer view. No setae. (A, C, D–F) Female (CRUST_JEGR/3916) from Málaga, 14.1 mm CW; B: Male (CRUST_JEGR/3918) from Málaga, 13.9 mm CW. Setae only indicated on some appendages. Scales 1 mm.

**FIGURE 4:**
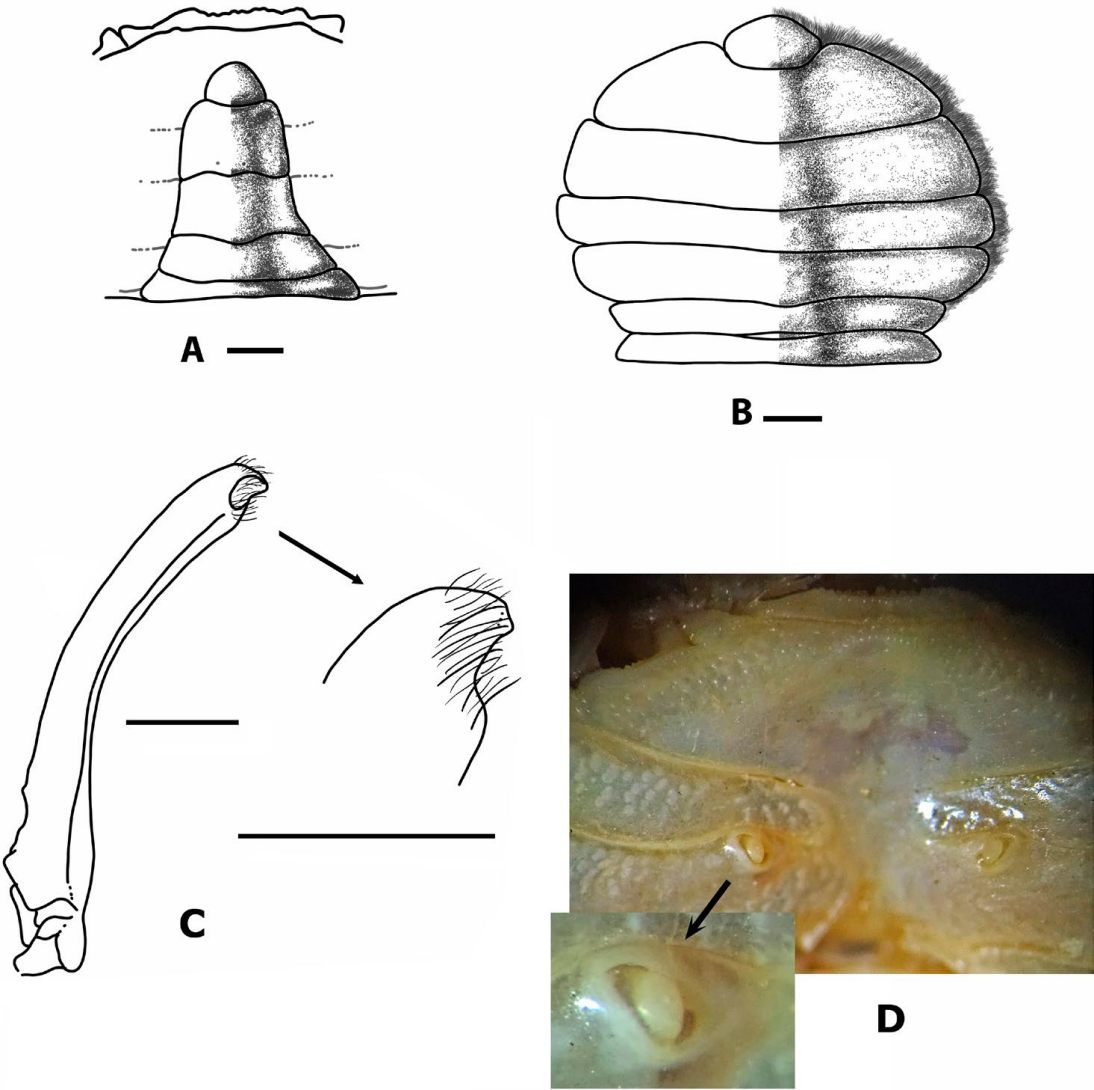
*Dudekemus atlanticus* n. gen., n. comb. (A) Male pleon, ventral view; (B) Female pleon, ventral view; (C) Left first pleopod of male, ventral view and detail of distal part; (D) Sternum and female vulva. (A) Male (CRUST_JEGR/3918) from Málaga, 13.9 mm CW; (B) Female (CRUST_JEGR/3916) from Málaga, 14.1 mm CW; (C) Male specimen (CRUST_JEGR/3918) from Málaga, 13.4 mm CW; (D) Female (CRUST_JEGR/3917) from Málaga, 14 mm CW. Setae only indicated on some appendages. Scales 1 mm.

**FIGURE 5:**
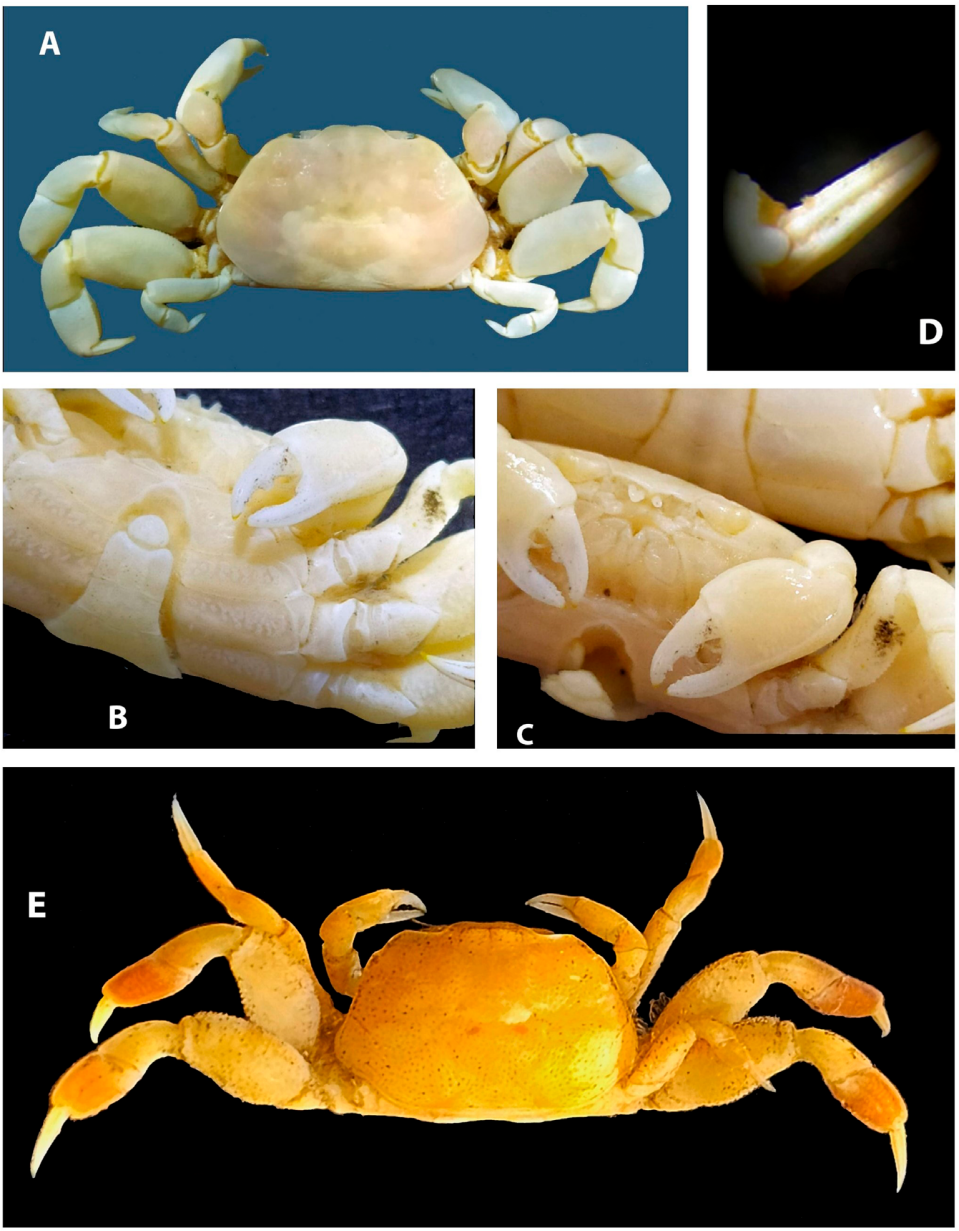
Photos of *Dudekemus atlanticus* n. gen., n. comb. (A, E) General view, dorsal view; (B) Pleon, ventral view; (C) Carapace, frontal view and chelipeds; (D) Detail of a dactylus. (A–D) Male specimen (CRUST_JEGR/3920) from Málaga, 13.0 mm CW; (E) Male specimen (RMNH.CRUS.D.57968) from Bruine bank, 7.52 mm CW.

**FIGURE 6:**
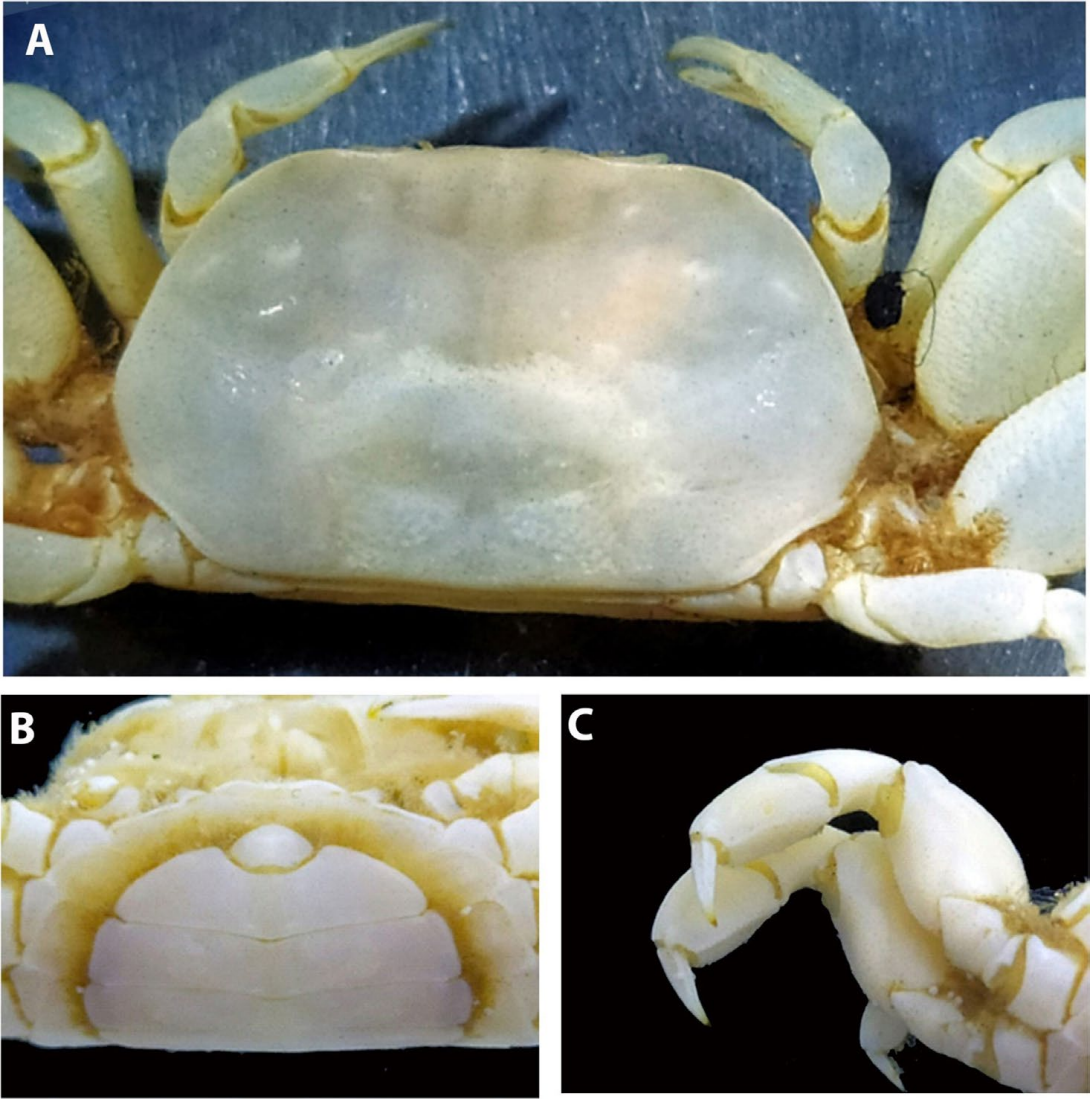
Photos of *Dudekemus atlanticus* n. gen., n. comb. (A) Carapace, dorsal view; (B) Pleon, ventral view; (C) P3 and P4 detail in antero‐ventral view. (A–C) Female from Málaga (CRUST_JEGR/3920), 13.6 mm CW.

**Genus *Dudekemus*** n. gen.

(Figures 2, 3, 4, 5, 6)

(urn:lsid:zoobank.org:act:5E79D9E3‐99ED‐4574‐80FF‐2AFFBBC8B370)

*Type Species*: *Dudekemus atlanticus* (Monod, 1933) n. comb. (previously *Asthenognathus atlanticus*), by monotypy.

*Diagnosis*: Carapace broadly trapeziform, markedly wider than long, more than 1.5 times wider than long; anterolateral margins entire, markedly divergent backwards, with anteroposterior lateral margin rounded, with sinuosity at this level in juvenile and medium‐sized specimens (up to 7.5 mm CW); oblique ridge present on posterolateral part of carapace; dorsal surface naked; broad front, width about 1/4–1/6 CW, slightly bilobed, slightly flexed downwards. Orbital cavity well‐defined externally; infraorbital ridge present. Antennal flagellum short, reaching distal part of the cornea or slightly extending beyond it. Third maxillipeds merus subquadrate; palp of normal size. Endostome without a longitudinal central ridge. ♂ P1 stout, palm high. P3 and P4 are the longest, with the merus, carpus and propodus distinctly broad; P5 is very reduced in size. ♂ and ♀ pleon with six free somites and telson. Lateral margin of so6 convergent towards distal part, slightly wider than proximal part of telson. G1 robust, slightly curved with a tuft of setae on apical part. G2 slender and small, distal part pointed. Vulva ovate, large, with a straight outer margin somewhat inclined on the inner surface.

*Etymology*: The name is dedicated to our dear friend and colleague Dr. Cédric d'Udekem d'Acoz for his excellent contributions to the knowledge of the decapod crustaceans of the Eastern Atlantic. Gender: masculine.

**FIGURE 7:**
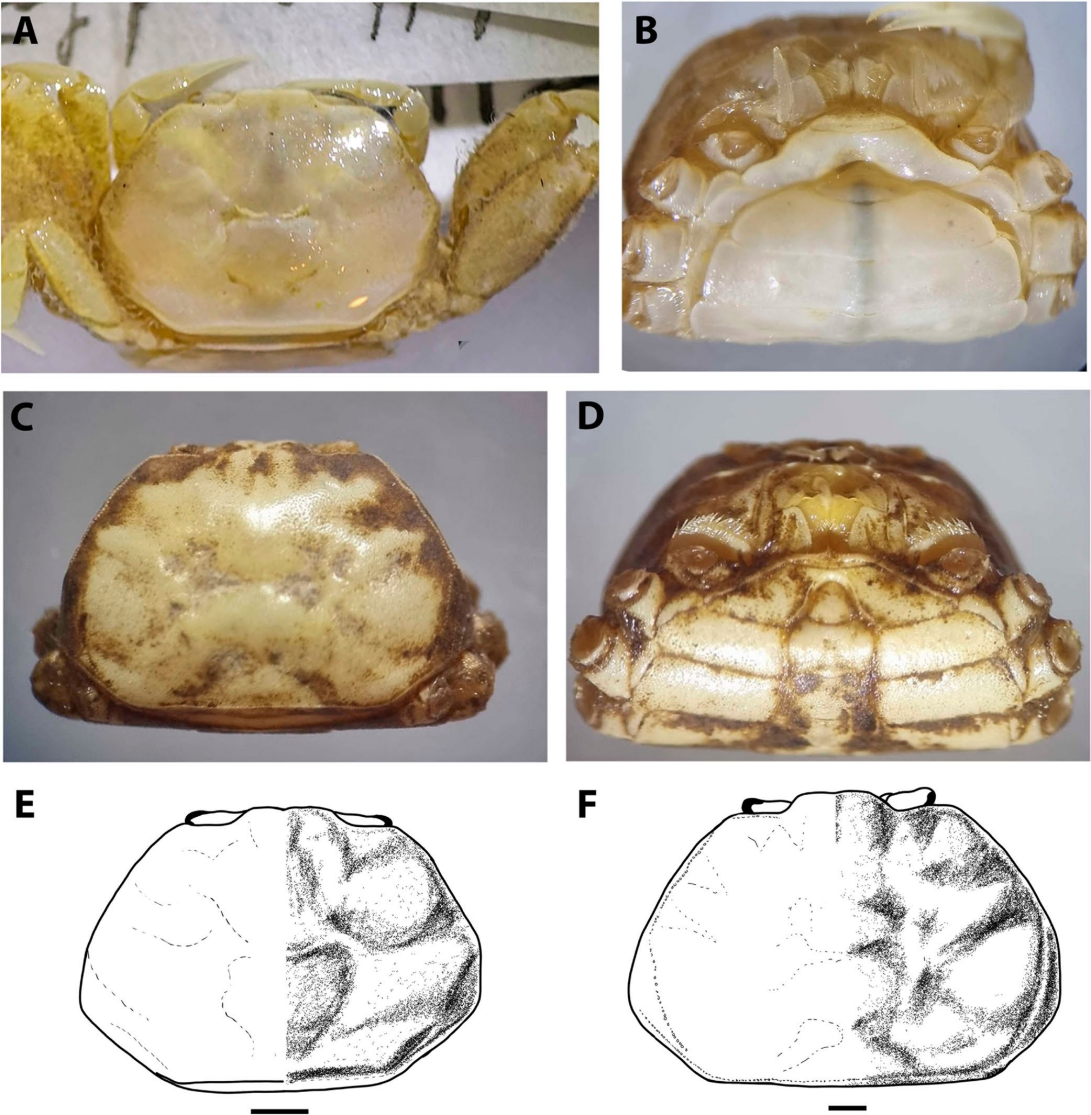
(A, B) Photos of the holotype of *Asthenognathus hexagonus*, ovigerous female, 7.7 × 5.7 mm CW × CL ZMUC_CRU_6704; (C, D) Photos of a male of *A. inaequipes*, 8.5 × 5.9 mm CW × CL, Crustacea_SMF_30931; (E) Scheme of the female holotype of *A. hexagonus* carapace; (F) Scheme of a male of *A. inaequipes* carapace. Scales 1 mm.

*Remarks*: This new genus (with a single species: *Dudekemus atlanticus* n. comb., previously classified within *Asthenognathus*) differs from the genus *Asthenognathus* (with two Indo‐Pacific species): *A. hexagonus* (Rathbun 1909; Jiang et al. 2007; Yang and Tang 2008; Wong et al. 2021) (Figure 7A,B,E) and *A. inaequipes* (Stimpson 1858; Jiang et al. 2007; Lee et al. 2010; Ko and Lee 2012) (Figure 7C,D,F) by several characters: a rather wider carapace more than one and a half times wider than long (less wide in *Asthenognathus*); the length of the frontal region (anterior part) is less than half the maximum width of the carapace; lateral margins of the sixth abdominal segment are convergent towards the distal part, which is slightly wider than the proximal part of the telson (*Asthenognathus* with parallel lateral margins so that the distal part is clearly wider than the proximal part of the telson).

Share with *Asthenognathus* some characters: the endostome without longitudinal central ridge; the merus of third maxilliped subquadrate (as *A. inaequipes*, longer in *A. hexagonus* ).

Another species previously included within *Asthenognathus* was *A. gallardoi* (today belonging to the genus *Gopkittisak* in the subfamily Gaeticinae), but it is very different because it shows the third maxilliped with the ischio‐merus joint obtuse and the anterior segments of the sternum with a longitudinal sulcus (Jiang et al. 2007; Naruse and Clark 2009, figure 1c; Komai 2011), while in *Dudekemus* the ischio‐merus joint is slightly inclined, transversal and without a longitudinal sulcus on the sternum; other characters are the more elliptical carapace and the existence of transverse ridges (not in *Dudekemus*).

***Dudekemus atlanticus*** (Monod, 1933) n. gen., n. comb.

(Figures 2, 3, 4, 5, 6).

*Synonyms*

*Asthenognathus atlanticus* Monod, 1933, p. 147, figures 6–9. Superseded combination (No *A. atlanticus* —Monod, 1956, figures 541–545).

*Tritodynamia atlantica*—Bocquet, 1963, p. 65–79, figures 1–6; Bocquet, 1965, pp. 407–418, figures 1–7. Superseded combination.

*Tritodynamea atlantica*—Glémarec & Hily, 1979, pp. 499–505. Superseded combination.

**EXAMINED MATERIAL. SYNTYPE**. MOROCCO • 1♂ (CW × CL: 3.5 × 2.5 mm, not 4.4 mm CW as cited in Monod 1933) MNHN‐IU‐2009‐889 (=MNHN‐B10572), Morocco (Atlantic Ocean), “Vanneau” Expedition, Stn. 17, 75 m, 33°57′N–07°05′W, 20‐07‐1923, Dollfus & Liouville coll., det. Monod Th. (as *Asthenognathus atlanticus*).

WESTERN SAHARA • 1 ♂ (2.8 × 2.2 mm), CRUST_JEGR/3927 in front of El Aaiún (Atlantic Ocean, border with MOROCCO), 13–30 m, 7 to 13‐03‐2024, Sergio Carlos García Gómez coll.

SPAIN • 1 ♀ (14.1 × 8.3 mm) CRUST_JEGR/3916 (the redescription and figures are mainly based on it) • 1 ♀ (14.0 × 8.3 mm) CRUST_JEGR/3917 (buccal frame) • 2 ♂ (13.9 × 8.9, 13.4 × 8.5 mm) CRUST_JEGR/3918 • 4 ♀ (13.1 × 7.0, 13.6 × 7.8, 14.1 × 8.3, 14.7 × 8.4 mm), 1 ♂ (13.3 × 8.2 mm) CRUST_JEGR/3919 • 1 ♂ (13.0 × 8.3 mm), 1 ♀ (13.6 × 8.0 mm) CRUST_JEGR/3920 (photos) • 1 ♂ (13.4 × 8.2 mm) CRUST_JEGR/3928 (16S: PQ248978); all these specimens from Málaga, thrown onto the Misericordia beach after storms, 1979–1981, soft bottom • 1 ♀ (10.4 × 5.6 mm) IEOCD‐AR13/845, Gulf of Cádiz, ARSA2013, Stn. 25, 24 m, 24‐02‐2013, 36.4813, • 1 ♂ (8.5 × 5.3 mm) CRUST_JEGR/3921.

FRANCE • 1 ♀ (13.2 × 7.7 mm), CRUST_ICMAN/3922, 1 ♂ (12.2 × 7.6 mm), CRUST_ICMAN/3923 (from J.C. Dauvin) Baie de Seine, Normandie, 09‐04‐2002.

NETHERLANDS • 1 ♂ (7.52 × 4.64 mm), RMNH.CRUS.D.57968, site code 006 from Brown Bank, Bruine Bank, North Sea, 52°21′58.0788″N, 03°27′47.664″ E to 52°21′55.5408″N, 03°27′51.3756″ E, EPSG425, dredge haul depth 32 m average, 24‐03‐2019 (photo).

*Etymology*: The name of the species refers, according to Monod (1933), to its known distribution area, the Atlantic Ocean.

*Diagnosis*: As for the Genus.

*Redescription*: This redescription and morphometric data primarily come from females and males from Málaga (Spain), Misericordia Beach, all adult specimens of large sizes (14.7 to 13.0 mm CW). Descriptive data can also be found, among others, in Bocquet (1963, mainly morphology; 1965, larvae), Ingle (1980), Faasse et al. (2021, morphology, distribution, ecology), Glémarec and Hily (1979, distribution, ecology), Jourde et al. (2012, distribution, ecology), Monod (1933) and in Pezy and Dauvin (2016, distribution, ecology).

Carapace (Figure 2A,B). Trapezoidal, wider than long, with females being slightly wider than males; adult CW/CL ratio in our large specimens: 1.69–1.87 ♀♀, 1.56–1.63 ♂♂ frontal margin and anterolateral margins with granular rim; bilobed front, slightly projected, directed downwards; external orbital angles not protruding, rounded, curving smoothly, merging with anterolateral margin; anterolateral and posterolateral margins approximately rectilinear, almost straight; anterolateral margin longer than posterolateral margin (approximately 1.6–1.8 times), diverging posteriorly to join posterolateral margin in gently curve beyond posterior half of carapace; posterior margin of carapace straight; faintly marked carapace regions, with two slightly marked frontal lobes (with median depression); gastric, hepatic, cardiac and intestinal regions poorly defined in adults; weakly marked grooves include 1 medially transverse one with a well‐marked cervical groove; no obvious latero‐posterior granular ridge in large adults. Eyestalks moderately long, less than twice as long as wide, curved upwards, with corneas somewhat narrower. Orbital cavity (Figure 2C) well developed, externally well delimited, internally open, communicating with the antennal basal article. Antennular pits wider than long, antennules folding transversely. Interantennular septum poorly defined medially. Antennal flagellum short, reaching distal part of cornea or slightly overreaching it. Suborbital region delimited by transverse, rectilinear ventral suture, this “infraorbital ridge” directed outwards. Epistome narrow, with a triangular central part, anterolateral edges slightly tuberculate and lateral parts concave. Pterygostomian region smooth, with smooth anterior margins or with some poorly developed tubercles. Buccal cavity (Figure 2C) with a curved anterior edge, sometimes the median part slightly more projected (slightly less pronounced than in *A. atlanticus* specimen of Monod (1933, figure 4)), and with arched lateral edges diverging backwards. Endostome without a longitudinal central ridge but with 2 latero‐longitudinal ones (not reach the anterior edge) that diverge backwards.

Third maxillipeds (Figure 2D) with wide separation between them when closed; ischium slightly longer than wide, convex inner edge with setae, concave outer edge with rounded basal projection, projected perpendicularly (90°) outwards, strongly setose; merus slightly longer than wide, with inner and outer edges curved, convex, slightly tuberculate, with distal angles (inner and outer) rounded, inner more pronounced, outer face with some slightly larger tubercles, with setae on both edges; longitudinal groove runs along outer faces of ischium and merus (latter somewhat more inclined, with numerous setae), closer to inner edges; ischio‐merus joint slightly inclined; palp: carpus > dactylus > propodus, with setae on lateral face; dactylus is tapering at the apex, with long setae at its distal part (but not reaching to sternum); distal part of exopod without flagellum, slightly exceeding half the length of the merus.

P1: in immature ♂ and ♀ similar, poorly developed; in adult ♂ more developed, rounded, clearly dimorphic; in ♀ (Figure 3A), both similar, not globose; proximal part of palm lower than distal, with dorsal edge curving downwards along posterior 2/5; anterior dorsal border of propodus slightly convex, ventral one concave medially, with strong subventral longitudinal carina running from almost proximal part of palm to distal part of fixed finger, reaching ventral edge medially; palmar L (measured medially: from distal to centre of inflexion)/palmar H ratio 1.1, dactylar/palmar lengths (medially measured) ratio 0.92; fingers of similar length, leaving a narrow central space between them; dactylus (mobile finger) with 5 or 6 rounded teeth, poorly developed, located from proximal zone to distal third (corneous part); fixed finger with 5–7 rounded teeth, poorly developed, located from proximal zone to distal, smaller towards distal part, with corneous claws. In adult ♂ (Figure 3B), chelipeds similar, well developed, globose; proximal palm lower than distal, dorsal edge curves downward in the posterior quarter; anterior dorsal border of propodus rounded, ventral almost rectilinear, slightly concave medially; palmar L (medially measured)/palmar H ratio 0.96–1, subventral longitudinal carina runs along the entire lower area of palm, less marked in the area of fixed finger; fingers of equal length, shorter than palm; dactylar/palmar lengths (medially measured) ratio 0.77, shorter, somewhat more curved than those of ♀, leaving a wide space between them; fingers with corneous claws; dactylus with strong tooth (single or double) medially (in anterior part of proximal half), and 1 or 2 very small towards distal part; fixed finger with very small rounded teeth (5–7) in distal half, proximal part concave, unarmed, internally (in sub‐dorsal area) with row of pits with setae; carpus smooth, inserting into the posterodorsal part of the propodus (palm); merus smooth, triangular in section, with convex external face, with distal part higher than proximal, dorsal area directed backward and downward; with a tuft of long anterodorsal setae.

Ambulatory legs (Figure 3C–F): P3 = P4 > P2 > P5. P2: slender (Figure 3C); merus rectangular in outer view, L (median part)/H ratio 1.85 ♀, 2.19 ♂, outer face smooth, with smooth, rectilinear dorsal edge, ventral edge slightly tuberculate, wider (curved) in proximal third, ventral face smooth, slightly inclined inwards, with poorly defined inner edge (without well‐defined row of tubercles, like on outer edge); propodus outer face smooth, with curved dorsal border (convex), almost rectilinear ventral one, slightly tuberculate, L (measured medially)/H ratio 1.07 ♀, 1.36 ♂, ventral face smooth, narrow, inclined towards interior, delimited externally by a row of tubercles (the tubercles of ventral edge, of outer face), antero‐internally with a poorly defined rim; dactylus slightly depressed, dorsally more convex, ventrally almost straight, more or less triangular in shape (in dorsal view), with longitudinal, rounded keel on lateral sides (external and internal), running from base (articular lobe of propodus) to apex, on dorsal and ventral sides there are 2 similar longitudinal keels, which delimit dorsal and ventral longitudinal central depressions, respectively; dactylus as long as propodus (0.9 ♀, 1.0 ♂).

P3 and P4 (Figure 3D,E): similar; merus, carpus and propodus broadened; length approximately the same as the maximum width of the carapace; merus: external face smooth, dorsal edge curved (convex), ventral also convex with maximum height towards the proximal third, L/H ratio as follows: P3 1.56 ♀, 1.6 ♂, P4 1.4 ♀, 1.7 ♂; carpus smooth; propodus external face smooth with curved dorsal edge, ventral edge more or less rectilinear, tuberculate, ventral face smooth, flat, delimited by 2 rows of tubercles (these are the rows of ventral edges of external and internal faces); propodus short, L/H ratio in large specimens (14 mm CW): P3 1.15 ♀, 1.4 ♂, P4 1.2 ♀, 1.15 ♂, in a small specimen (♂ of 7.8 CW) L/H ratio of (P3 1.5, P4: 1.25); dactylus as in P2, depressed, more or less triangular in shape (in dorsal view), with a longitudinal, rounded keel or ridge on lateral sides (outer and inner), running from base (from articular lobe of propodus) to apex, on dorsal and ventral sides with 2 similar longitudinal keels, which delimit dorsal and ventral longitudinal central depressions, respectively; dactylus/propodus lengths ratio of P3 1.1 ♀, 0.8 ♂, P4 1.1 ♀, 1.1 ♂.

P5 (Figure 3F): reduced; merus smooth with rectilinear dorsal edge, with setae, curved ventral edge, slightly tuberculate, broader in proximal third; maximum length‐to‐height ratio is 1.85 ♀, 2.25 ♂; carpus smooth, propodus with slightly curved dorsal edge, tuberculate, curved ventral edge with relatively long setae, ventral face smooth, inclining inwards, tapering towards proximal part, delimited externally by row of tubercles on ventral edge of external face (in ventral view, looks like keel), L/H ratio 1.0 ♀, 1.2 ♂; dactylus as in P3, P4, dactylus/propodus lengths ratio 0.9 ♀, 1.0 ♂.

The anterior edge of the sternum is rounded and tuberculate, with 1 or several more developed tubercles in the middle. Sternites 1 and 2 fused, without a longitudinal sulcus between them.

Pleon of ♂ and ♀ with 6 distinct somites and telson; triangular in adult ♂ (Figure 4A), with distal part of the so6 only slightly wider than the basal part of the telson; rounded in adult ♀, large (Figure 4B), narrower in juveniles (as those figured by Monod 1933, figure 9A), Bocquet (1963) notes ♀♀ 11.4 mm CW show a less rounded prepubertal‐type pleon.

G1 (Figure 4C): robust, slightly curving from the median part (inflexion closer to the proximal half), outer face flat, with a row of short setae along the entire concave edge, distal part with a tuft of short setae, with a small external projection (cf. Bocquet 1963, figure 6.1; Figure 4C here). G2: slender, small, G1/G2 ratio 5–6.5 (4.3 in Bocquet 1963), with a somewhat pointed apex.

Vulva: oval‐shaped, large, with operculum, occupying the upper third of ♀ s6, with straight and inclined outer margins from outside (anterior) to inside (posterior) (Figure 4D).

*Variation*: The CW/CL ratio of the small ♂ specimen (7.5 mm CW, ratio 1.62) from Bruine Bank (RMNH.CRUS.D.57968) is distinctly less than in adults, but this is probably associated with its immaturity. While the anterolateral margin in large ♂♂ is unarmed, the area may show a sinuosity (see Monod 1933, figure 6; Pezy and Dauvin 2016, figure 1A; Faasse et al. 2021, figure 2), and this is also evident on the smallest ♂ specimen from Bruine Bank (7.5 mm CW, RMNH.CRUS.D.57968). Bocquet (1965) described a small tooth present in megalopas obtained (from Térénez, Bretagne, France) but this structure diminishes with development and disappears in adults. There is no obvious granular ridge on the latero‐posterior part in the adults examined (if present, it runs obliquely backwards from each posterior part of the anterolateral edge), but it is evident in juvenile specimens reported by Monod (1933, figures 6, 8E; from Morocco) and Bocquet (1965, figure 1; from Roscoff), and it is also present in the young Bruine Bank specimen (RMNH.CRUS.D.57968) (Figure 2B).

*Maximum Sizes*: Female 14.7 mm, male 13.9 mm CW (specimens from Málaga, Spain). Jourde et al. (2012) cited that specimens from 2.2 to 4.8 mm are sexually undeterminable.

*Colouration*: Ochre‐beige dorsal carapace, transitioning to a reddish‐brown on the hepatic and posterior branchial regions. These areas may also appear bluish‐grey, while the lateral carapace edges, front and intestinal region are often yellowish or bluish‐white. Pereiopods P2–P5, especially P3 and P4, exhibit a characteristic red‐wine band on the distal propodus (Bocquet 1963). Faasse et al. (2021) also observed this coloured band on the distal half of the carpus of the second and third pereiopods.

Small individuals, and some medium‐sized ones, have a uniform ferruginous yellow colour, which can darken to brown in rare cases. Very young crabs are uniformly sandy‐coloured (Bocquet 1963). Faasse et al. (2021) also observed that second to fifth pereiopods are held between parallel tangents, running along the anterior and posterior boundaries of the carapace. Both descriptions pointed out that coloration changes across developmental stages (Bocquet 1963; Faasse et al. 2021). Some colour photographs could also be found in Noël and Ziemski (1933) in DORIS (2020); Asturnatura https://www.asturnatura.com/especie/asthenognathus‐atlanticus.

*Habitat*: Always on soft bottoms. On muddy sand with the urchin *Brissopsis lyrifera* (Forbes, 1841), Morocco, 70 m (Monod 1933). On muddy sands, seagrass beds of *Zostera*, commensal of the polychaete *Neoamphitrite edwardsii* (Quatrefages, 1866) (as *Amphitrite edwardsii* ), Roscoff, France, intertidal zone (Bocquet 1963). On muddy sediments, muddy fine sands, sandy muds and mud; commensal of *N. edwardsii* , also, in association with the cnidarian (cerianthid) *Cerianthus membranaceus* Spallanzani, 1784, the sipunculid *Sipunculus nudus* Linnaeus, 1766, and, mainly, with the holothurian *Labidoplax digitata* Montagu, 1815, some specimens in stomachs of *Raja clavata* Linnaeus, 1758, English Channel, Bay of Biscay, intertidal to 50 m (Glémarec and Hily 1979). On mud, muddy sand and sand, eastern English Channel, France, 7–25 m (Jourde et al. 2012). On muddy, fine sand and coarse sands, commensal with the polychaete *Chaetopterus variopedatus* (Renier, 1804) (31% of *Chaetopterus* tubes contained one crab), some in stomachs of *Raja clavata* , eastern English Channel, 12–25 m (Pezy and Dauvin 2016). On sand with shell grit, together with the mud shrimp *Callianassa subterranean* (Montagu, 1808) and/or *Gilvossius tyrrhenus* (Petagna, 1792) and the urchin *Echinocardium cordatum* (Pennant, 1777), Bruine Bank, Netherlands, 32 m (Faasse et al. 2021).

*Depth Range*: Intertidal to 70 m.

*Distribution*: Atlantic waters: from the North Sea (Bruine Bank, Netherlands), Normandy, the English Channel, NW France, Spain to Morocco (between Mohammedía (Fedhala) and Rabat, in front of Skrirat) and Western Sahara, in front of El Aaiún; and the West Mediterranean Sea (Monod 1933; Noël and Amouroux 1977; Faasse et al. 2021). Guinot and Ribeiro (1962) cited the species in Angola, but see Section 4.

*Remarks*: *Dudekemus atlanticus* n. comb. (previously within *Asthenognathus*) differs from *A. hexagonus* (Rathbun 1909; Yang and Tang 2008; Wong et al. 2021) and *A. inaequipes* (Stimpson 1858; Lee et al. 2010; Ko and Lee 2012) in: (1) a rather wider carapace, more than one and a half times wider than long; (2) the length of the frontal region (anterior part), less than half the maximum width of the carapace; (3) the meeting zone of the anterolateral and posterolateral edges of the carapace, rounded (hexagonal in *A. hexagonus* , but not in *A. inaequipes*); (4) merus, carpus and propodus of P3 and P4 clearly broadened (not in *A. hexagonus* in which are narrower, elongated, but broadened at in *A. inaequipes*); (5) lateral margins of the sixth abdominal segment convergent towards the distal part, which is slightly wider than the proximal part of the telson (*Asthenognathus* with parallel lateral margins so that the distal part is clearly wider than the proximal part of the telson).

These three species possess an endostome without a longitudinal central ridge, unlike other varunid species such as the Gaeticinae *Paranotonyx curtipes* Nobili, 1906 (Ng and Davie 2024).

The morphological differences between *D. atlanticus* and the species of *Schubartus* are detailed below, after their description in the remarks section.

The latero‐posterior granular ridge of the carapace is also present in other African specimens illustrated by Monod (1956, figures 541, 541bis) as *Asthenognathus atlanticus*, but they belong to another species (described below), so this feature is also not useful for distinguishing them. On walking legs: the male juvenile described by Monod (1933, figure 9c–f) exhibits more slender legs, with apparently narrower dactylus and longer propodus than in our medium‐large European specimens (which resemble those of *Schubartus*'s new species, described below). Bocquet (1963) mentioned that P2–P5 are gracile in juveniles, but after puberty, the propodus, carpus and merus of P3 and P4 undergo a marked broadening. G1 of a small specimen from Morocco illustrated by Monod (1933, figure 9b) lacks detailed features and presents a narrowed and curved distal part (different from that of our and Bocquet's (1963 specimens)), but it is probably due to its small size and immaturity. Later, Monod (1956, figure 545) drew the gonopod of a specimen from Senegal, as *Asthenognathus atlanticus*, but it appears very different from the taxa we have studied; it could be another undescribed species.

**FIGURE 8:**
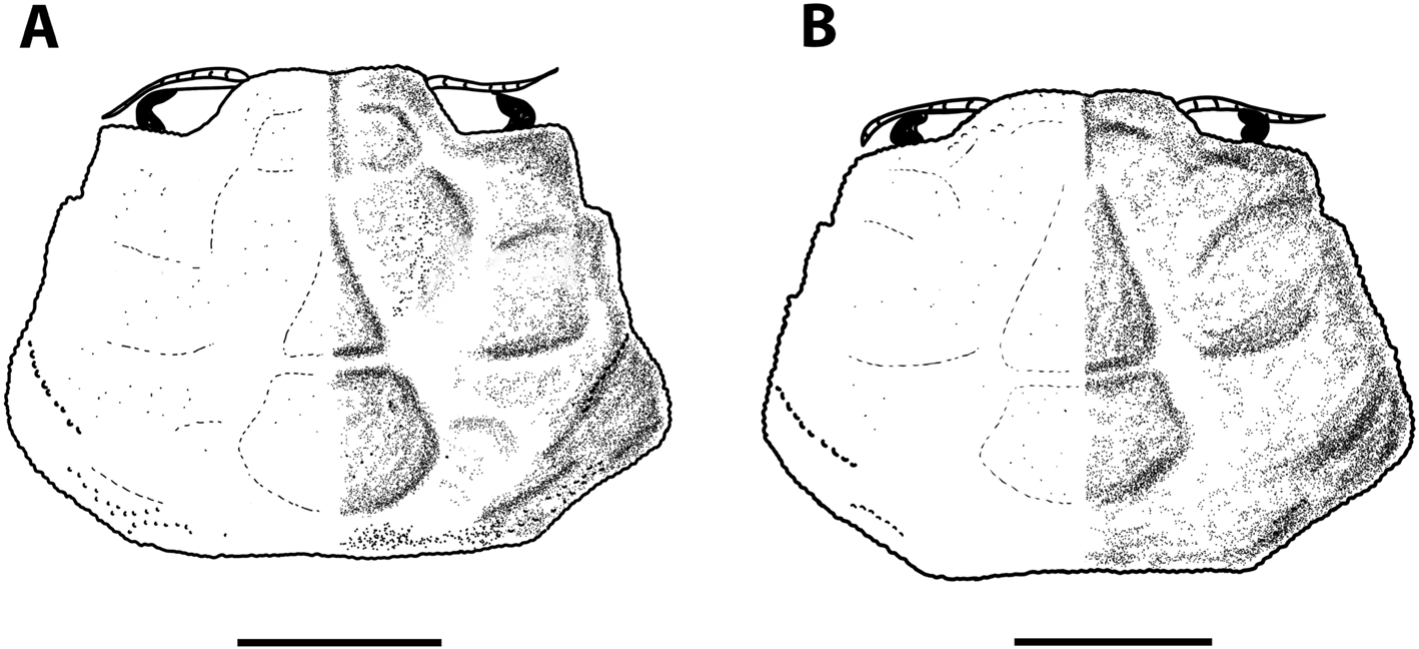
(A) Carapace of male juvenile of *Dudekemus atlanticus* n. gen., n. comb. of 3.5 mm CW from Morocco (Syntype of *Asthenognathus atlanticus* Monod, 1933; MNHN‐IU‐2009‐889, =MNHN‐B10572); (B) Carapace of male juvenile of *Schubartus ngankeeae* n. gen., n. sp., 4.2 mm CW, from Mauritania (RMNH.CRUST D40008‐3). Scales 1 mm.

**FIGURE 9:**
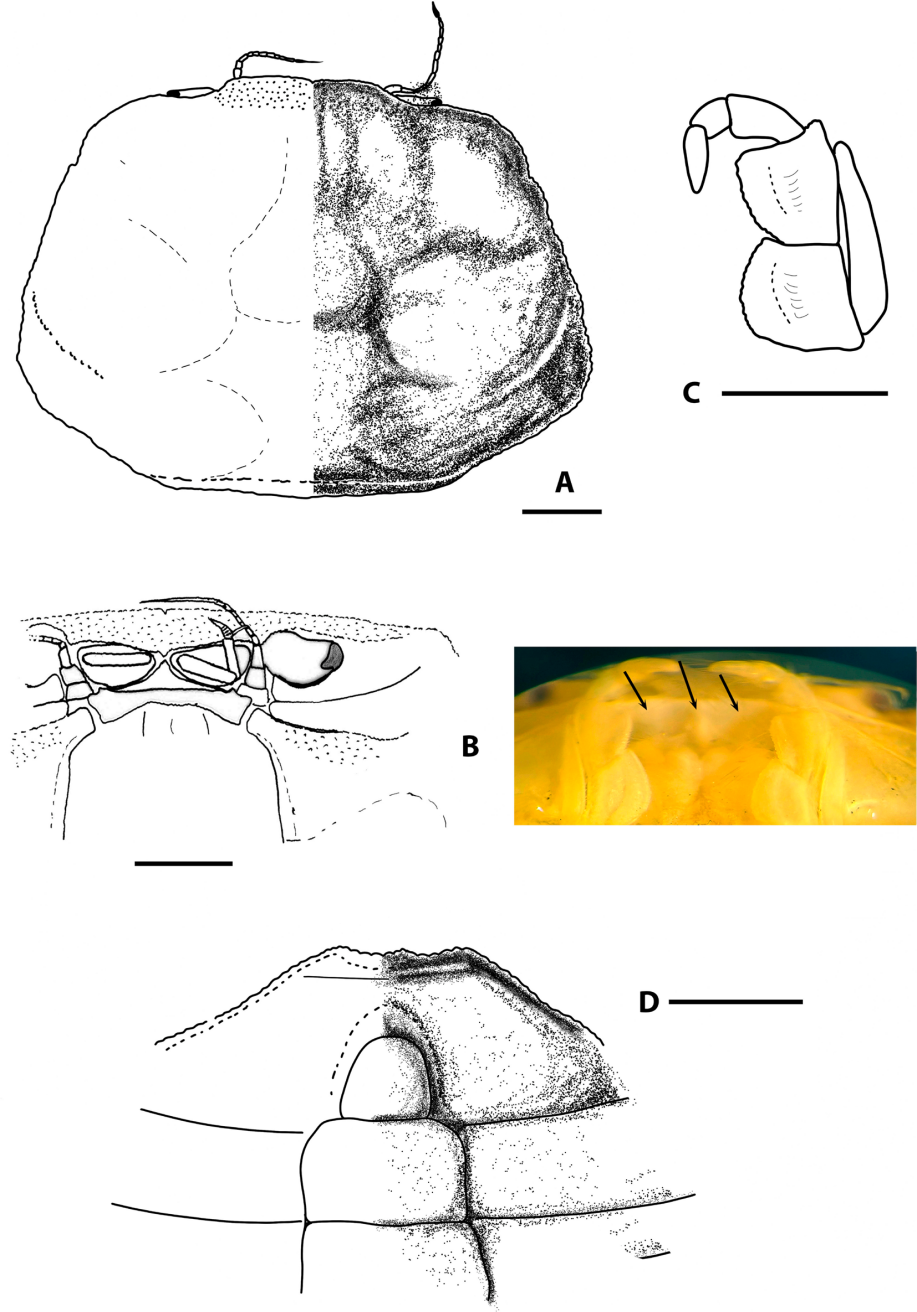
*Schubartus mauritanicus* n. gen., n. sp. (A) Carapace, dorsal view; (B) Partial view of antero‐ventral region, orbital cavity, buccal frame and endostome (figure and photo); (C) Left third maxilliped, ventral view; (D) Anterior part of sternum and male pleon, ventral view. Male holotype (D40008‐1 RNMH), 7.2 mm CW, from Mauritania. Scales 1 mm.

**FIGURE 10:**
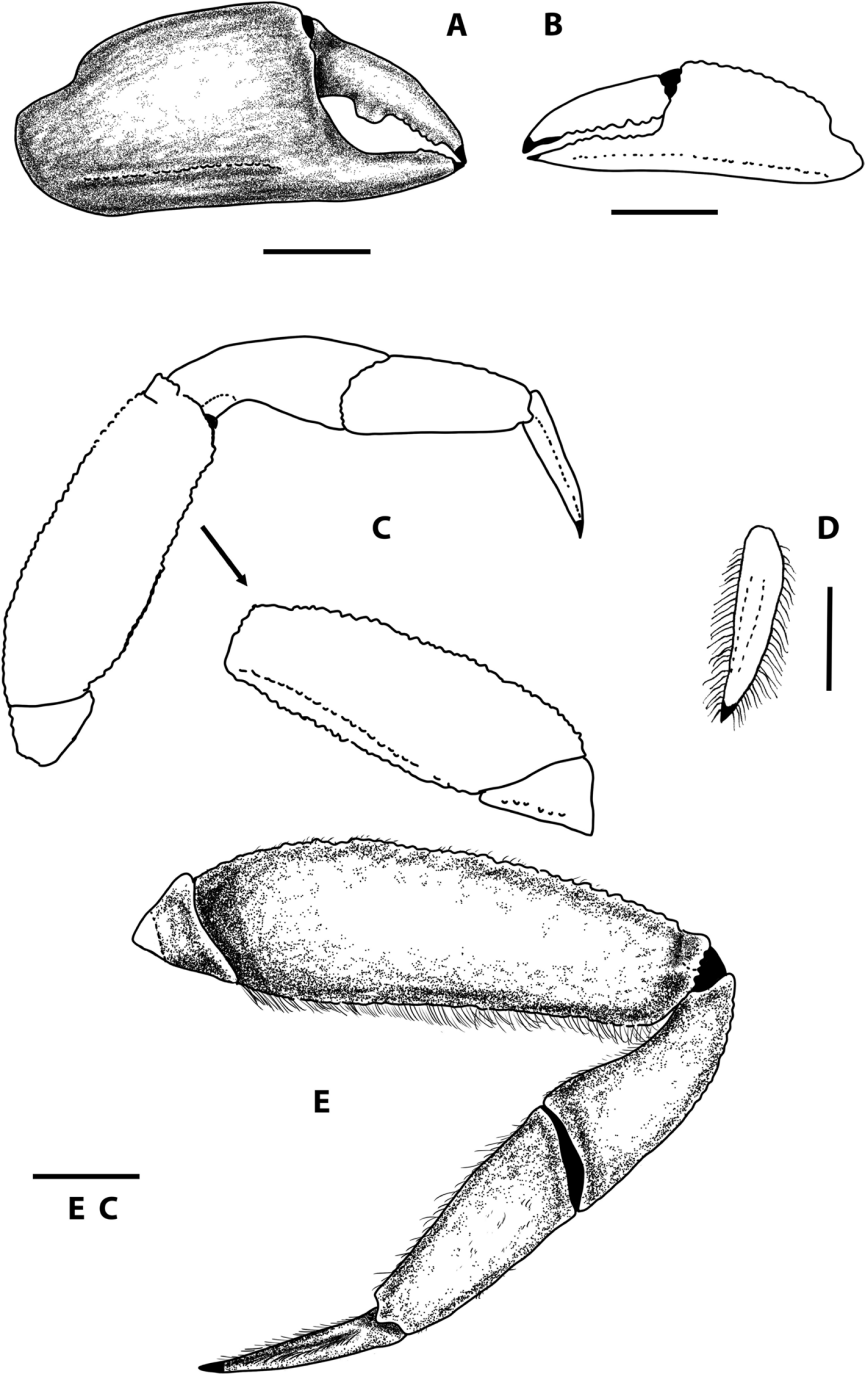
*Schubartus mauritanicus* n. gen., n. sp. (A) Right cheliped, male, outer view; (B) Left cheliped, female, outer view; (C) P2 right, outer view, detail of right merus inner view; (D) Dactylus of P2, ventral view; (E) P3 right, outer view. (A, C, D, E) Male holotype (D40008‐1 RNMH), 7.2 mm CW; (B) Female paratype (D40008‐2 RNMH), 8.6 mm CW. All from Mauritania. Setae are only indicated on some appendages. Scales 1 mm.

**FIGURE 11:**
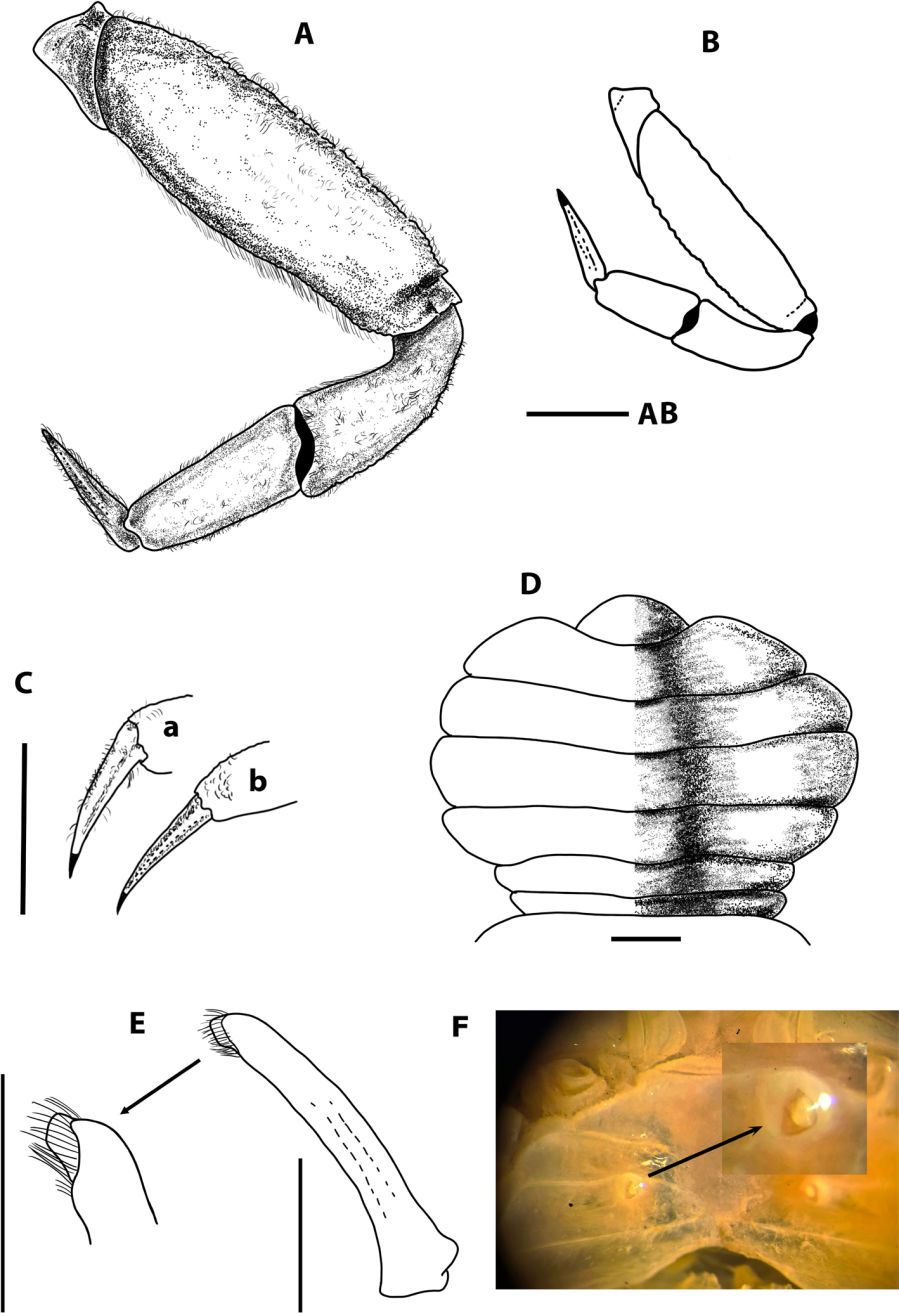
*Schubartus mauritanicus* n. gen., n. sp. (A) P4 right, male, outer view; (B) P5 right, outer view; (C) Dactylus of the right P3 (a) and P4 (b) of the female; (D) Pleon of adult female, ventral view; (E) Right first pleopod of male, ventral view and detail of distal part; (F) Sternum and female vulva. (A, B, E) Male holotype (D40008‐1 RNMH), 7.2 mm CW; (C, D, F) Female paratype (D40008‐2 RNMH), 8.6 mm CW. All from Mauritania. Setae only indicated on some appendages. Scales 1 mm.

**FIGURE 12:**
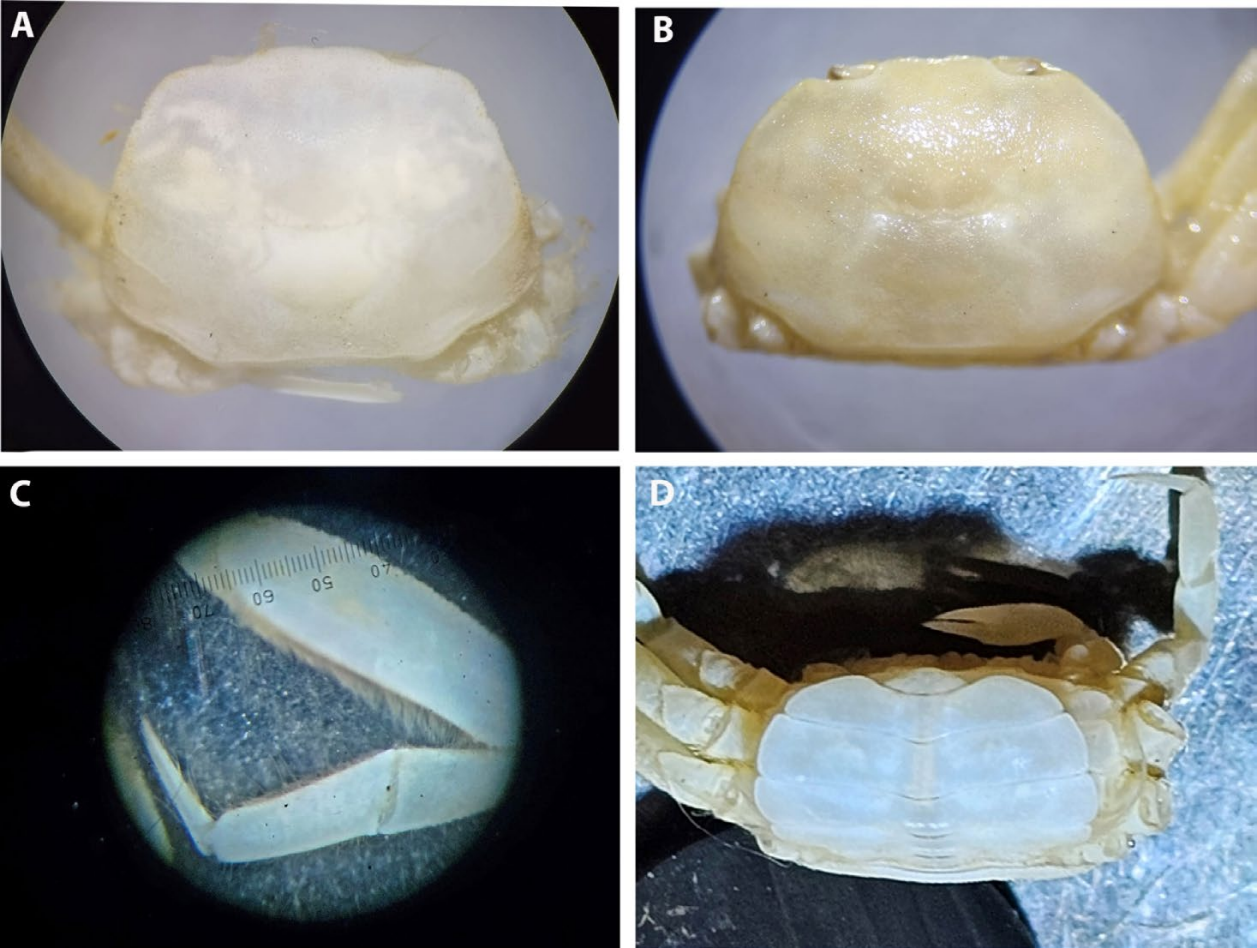
Photos of *Schubartus mauritanicus* n. gen., n. sp. (A) Male holotype carapace (D40008‐1 RNMH); (B) Female paratype carapace (D40008‐2); (C) Left P4 from the male holotype (D40008‐1 RNMH); (D) Pleon from the female paratype (D40008‐2 RNMH). All from Mauritania.

***Schubartus*** n. gen.

(Figures 8B, 9, 10, 11, 12).

(urn:lsid:zoobank.org:act:31395B2F‐DF86‐4FD2‐9098‐BC6C339FAD0A)

*Type Species*: *Schubartus mauritanicus* n. gen., n. sp., by present designation. Gender masculine.

*Diagnosis*: Carapace broadly trapeziform, markedly wider than long, < 1.5 times wider than long (1.3–1.5); external orbital angles rounded, not protruding; anterolateral margins entire, divergent backwards; in adults, no distinct anterolateral tooth, with sinuosity at this level; posterolateral margin shorter than anterolateral margin; with oblique granular ridge on dorsal posterolateral part of carapace, running backwards; faintly marked carapace regions, with evident transverse cervical and gastro‐cardiac grooves; broad front, slightly bilobed with median depression, gently flexed downwards. Distal part of the cornea resting outside the orbital cavity, on a long transverse orbital depression that extends latero‐ventrally to the lateral zone, with outer border of orbital cavity hidden by cornea. Orbital area ventrally delimited by infraorbital ridge‐groove, running laterally upwards. Antennal flagellum relatively long, extending beyond distal part of cornea (approximately twice the length of the ocular peduncles). Epistome triangular and short, with the median part of the posterior margin triangular. Triangular pterygostomial region, with tubercles on the anterofrontal area. Endostome with a longitudinal central ridge or projection. Third maxillipeds separated by a wide hiatus. ♂ P1 stout, with a high palm, fingers less long than the palm, dactylus with a strong protruding tooth on the inner margin. P2‐P5 elongated, slender; P3 and P4 approximately 1.5–1.6 times longer than maximum carapace width; P5 is very reduced. Adult ♂ with triangular pleon, so6 having more or less straight lateral edges, tapering distally to meet a narrower, semicircular telson. G1 robust, with uniform width along length, curved outwards, with the distal area surrounded by a crown of setae. G2 slender and small. Vulva ovate, large, with operculum occupying upper third of ♀ s6, inner surface slightly inclined towards median line.

*Etymology*: The name of this genus is dedicated to our colleague and dear friend Christoph D. Schubart who recently passed away, in recognition of his great contributions to carcinology, especially to the Thoracotremata systematics. Genre masculine.

*Material Examined*: The examined material of this genus is mentioned at the beginning of the descriptions of each of the two new species. However, there are very small or legless specimens in which DNA data could not be obtained, so the morphological identification of these specimens is uncertain, and they cannot be assigned with certainty to either species of *Schubartus*. These are: MAURITANIA • 2 ♂♂ (6.5 × 4.7, 4.2 × 3.3 mm) and 1 ♀ (6.6 × 4.6). RMNH.CRUS. D40008 (as *Asthenognathus atlanticus* Monod, 1933, det. C.H.J.M. Fransen, 1991) “TYRO” Mauritania‐II Expedition 1988, Stn. MAU.101 Mauritania, off Banc d'Arguin, 19°43′N–16°59′W, 61–72 m., 16‐06‐1988. Sticky grey mud with some sand and shell gravel, spider crabs (*Inachus* sp., *Macropodia* sp.), bivalves, gastropods, tubeworms, hermit crabs and flatfish. 2.4 m Agassiz trawl. • 2 ♂♂ (4.3 × 2.9 mm, broken carapace) and 2♀♀ (5.25 × 3.4, 4.9 × 3.0 mm). MNHN‐IU‐2009‐902 (=MNHN‐B10588) (as *Asthenognathus atlanticus* Monod 1933). Mauritania, N. Baie de Cansado, Banc de Ardent, 14 m. 20‐06‐1953, coll. Marche‐Marchad; • 1 ♂ (8.0 × 5.5 mm). RMNH.CRUS. D39699 (as *Asthenognathus atlanticus* Monod, 1933, det. C.H.J.M. Fransen, 1991), “CANCAP III” Expedition, Stn. 3121. Off Mauritania 20°22′N, 17°40′W. 210 m. 28‐10‐1978. Sandy clay with shell and *Lanice* tubes. 2.44 m Agassiz Trawl.

IVORY COAST • 1 ♀ (4.6 × 3.3 mm). MNHN‐IU‐2009‐897 (=MNHN‐B24084) (as *Asthenognathus atlanticus* Monod, 1933). Det. Naruse. T. Cote d'Ivoire. Golfe de Guinée. «Guinean Trawling Survey II» Expedition, Stn. Dragage 14.04°52.30′N, 05°57.30′W. 70 m. 02‐04‐1964. Hard substrates with calcareous algae.

*Remarks*: The main morphological differences between the monotypic genus *Dudekemus* n. gen. and *Schubartus* n. gen. (see also Remarks of the *S. mautitanicus* n. gen., n. sp.) are: *Schubartus* n. gen. (1) has a broadly trapeziform carapace, markedly wider than long, but < 1.5 times (1.3–1.5) (in *Dudekemus n. gen*. more than 1.5); (2) the anterolateral margins are less divergent backwards compared to those of *D. atlanticus* ; (3) the cornea overlaps the outer border of the orbital cavity, hiding it and reposes on a long transverse orbital depression, while in *Dudekemus* n. gen. the entire eyestalk is within the orbital cavity; (4) the antennal flagellum is relatively long and extends well beyond the distal part of the cornea (about twice the length of the eyestalks); in *D. atlanticus*, it reaches or very slightly exceeds it; (5) the endostome has a longitudinal central ridge (not in *D. atlanticus* ); (6) P3 and P4 have a relatively narrower merus, carpus, and propodus (broaderned in *D. atlanticus* ); (7) the ♂ so6 has straight lateral edges, tapering distally to meet the telson, which is proportionately narrower (in *D atlanticus* these ones are convergent towards the distal part, which is slightly wider than the proximal part of the telson).

Juvenile specimens of *D. atlanticus* present (as mentioned above) a morphology somewhat different from that of the adult (Monod 1933, figures 8, 9; Figure 8A here). These juveniles can be distinguished from the juveniles of *Schubartus* n. gen. (*S*. *ngankeeae* n. sp., n. gen.) (Figure 8B) by the absence–presence of a longitudinal central ridge on the endostome (a generic level difference), as well as a carapace that has more concave lateral margins (in *S. ngankeeae* n. sp., n. gen., they are more or less rectilinear or very slightly concave) and a straight posterior orbital rim with the external part somewhat directed forwards (the external angle is angular or not), while in *Schubartus* n. gen. (*S. ngankeeae* n. sp., n. gen.), it is slightly convex, curving slightly backwards.

*Schubartus* n. gen. differs from the two species of *Asthenognathus*, as it lacks the longitudinal medial ridge of the endostome. In addition, *A. hexagonus* has a clearly hexagonal carapace, the fingers of chelipeds are as long as the palm (in females, much longer), and the dactylus of males has a broad but not very protruding tooth on the inner margin. In *Schubartus* n. gen., the carapace is more trapezoidal, the finger is shorter than the palm, and the male dactylus has a strong and protruding tooth on the inner margin; in *A. inaequipes*, P3 and P4 have broadened merus (not narrower as in *Schubartus* n. gen.).

***Schubartus mauritanicus*** n. gen., n. sp.

(Figures 9, 10, 11, 12)

(urn:lsid:zoobank.org:act:0157771E‐B7E6‐4FD6‐9566‐67B7FCD481AB)

*Asthenognathus atlanticus*—Monod, 1956 (specimens from Mauritania figure 541 and Senegal figure 541 bis).

**EXAMINED MATERIAL. HOLOTYPE**. MAURITANIA • 1 ♂ (CW × CL: 7.25 × 5.4 mm), RMNH.CRUS. D40008‐1 (as *Asthenognathus atlanticus* Monod, 1933, det. C.H.J.M. Fransen, 1991) “TYRO” Mauritania‐II Expedition 1988, Stn. MAU.101, off Banc d'Arguin, 19°43′N, 16°59′W, 61–72 m., 16‐06‐1988. Sticky grey mud with some sand and shell gravel, spider crabs (*Inachus* sp., *Macropodia* sp.), bivalves, gastropods, tubeworms, hermit crabs, flatfish. 2.4 m Agassiz trawl.

**PARATYPE**. MAURITANIA. • 1 ♀ (8.6 × 5.8 mm ovigerous). RMNH.CRUS. D40008‐2 (as *Asthenognathus atlanticus* Monod, 1933, det. C.H.J.M. Fransen, 1991) same data as holotype.

*Diagnosis*: As for genus, in this species the carapace regions are less pronounced in adults, particularly the lobes or projections of the anterolateral margin, with a distinct transverse cervical groove in the median part. P3 and P4 are the longest, with the merus, carpus and propodus relatively narrower; the merus length‐to‐height ratio is 3.0–3.3 ♂, 2.4–2.6 ♀. Vulva oval‐shaped, large, with the outer margin more or less rectilinear, and the inner surface is gently inclined.

Carapace broadly trapeziform, wider than long, < 1.5 times; anterolateral margins entire, with sinuosity at this level; posterolateral margin shorter than anterolateral margin; carapace regions less pronounced in adults, particularly lobes or projections of anterolateral margin, with distinct transverse cervical groove in median part; with oblique granular ridge on dorsal posterolateral part of carapace; broad front, slightly bilobed with median depression, gently flexed downwards. Distal part of the cornea resting outside the orbital cavity, on a long transverse orbital depression that extends latero‐ventrally to the lateral zone. Orbital area ventrally delimited by infraorbital ridge. Antennal flagellum relatively long, extending beyond distal part of cornea (approximately twice the length of ocular peduncles). Epistome triangular, short. Triangular pterygostomial region, with tubercles on the anterofrontal area. Endostome with a longitudinal central ridge or projection. Third maxillipeds with wide separation between them when closed. P3 and P4 are the longest, with the merus, carpus and propodus relatively narrower, the merus length‐to‐height ratio is 3.0–3.3 for ♂, 2.4–2.6 for ♀; P5 is very reduced. ♂ pleon in pagoda. G1 robust, with uniform width along length, curved outwards, with the distal area surrounded by a crown of setae. Vulva oval‐shaped, large, with outer margin more or less rectilinear, inner surface gently inclined.

*Etymology*: The name refers to the geographical area where the holotype was collected.

*Description*: Carapace (Figure 9A): trapezoidal, wider than long, somewhat more in female than in male (Figure 12A,B), CW/CL 1.34 ♂, 1.50 ♀, maximum width located towards the posterior part of the carapace (about three‐quarters length); carapace with tuberculated edges (somewhat more pronounced in smaller specimens); bilobed front, very little projected, directed slightly downwards, with frontal dorsal surface tuberculated; external orbital angles not protruding, rounded, curving smoothly, merging gradually with the anterolateral margin, anterolateral and posterolateral margins more or less rectilinear almost straight; anterolateral margin clearly longer than posterolateral margin, diverging posteriorly to join with the posterolateral margin in a curve on the posterior of the carapace; adults with no distinct anterolateral tooth, with small depression or sinuosity at this level (in ♀, outer orbital edge more angular and anterolateral part more rounded, practically without sinuosity); posterior margin of the carapace almost straight, with tuberculated anterior transverse ridge; faintly marked carapace regions, with 2 poorly defined frontal lobes (with slight median depression), with weakly marked grooves, with transverse one, more evident, cervical, medially; 2 tuberculate dorso‐posterolateral ridges run obliquely backwards from each posterior part of the anterolateral edges. Eyestalks moderately long, less than twice as long as wide, somewhat conical, curved upwards, wider basally and narrower distally, in the corneal zone; rounded cornea overlaps the orbital cavity, hiding the outer edge, resting on a long transverse orbital depression (Figure 9B) that extends latero‐ventrally to the edge of the anterolateral boundary; orbital cavity open internally, so that eyestalks are in contact with the basal segment of the antenna. Antennules folding transversely, with well‐defined interantennular septum. Antennal flagellum relatively long, extending well beyond the distal part of the cornea (twice the length of eyestalks), reaching the anterolateral edge. Suborbital region delimited anteriorly by transverse, rectilinear, outwardly directed ventral suture. Epistome short, triangular, narrow, with posterolateral parts concave. Triangular pterygostomial region with tuberculate anterior edge; tubercles present on the posterior edge of the anterior sclerite and anterofrontal area. Buccal cavity with a uniformly curved anterior edge with arched lateral edges that slightly diverge backwards. Endostome with a longitudinal central ridge and two inconspicuous latero‐longitudinal ones that do not reach the distal edge of the buccal frame (Figure 9B).

Third maxillipeds (Figure 9C): with wide separation between them when closed; ischium practically as wide as long, wider proximally, inner margin slightly convex, lobed, with setae, outer margin slightly concave, without a well‐developed basal lobe projected outwards, with a longitudinal groove in the center of the segment; merus as long as wide, distally widened, with rounded upper‐lateral angles, outer slightly more protruding, denticulate, inner margin curved, convex, with setae and tuberculate, outer margin with proximal half straight or somewhat concave and distal convex; ischio‐merus joint slightly inclined, almost straight; palp: carpus > dactylus > propodus, with setae on lateral faces; dactylus finger‐shaped, with long setae; distal part of exopod (without flagellum) almost reaching distal part of merus of endopod.

P1: right and left similar in ♂, globose (Figure 10A); outer face of propodus convex, smooth, with some short setae, with longitudinal row of tubercles in the basal quarter, extending from the basal part of the palm to almost the base of the fixed finger (curving towards distal part, e.g., in small specimens), with setae extending through the fixed finger, dorsal edge curved, convex, with some little tubercles, especially in the basal area, not very marked, smooth, convex inner face; postero‐ventral‐internal area, at the joint with the carpus, with tubercle with 3–4 obvious granules; palm of propodus (length‐to‐height ratio, measured medially) 1.5 times longer than high; fingers of similar length, fixed one curved distally upwards, with 7 or 8 small distal tubercles on the inner edge, ending in corneous claw; dactylus, with dorsal edge slightly curved, with strong inner quadrangular tooth in the anterior part of the proximal half, 5 or 6 small tuberculated teeth in the anterior part, with corneous distal apex; carpus convex, smooth, antero‐ventral edge tuberculate; merus with narrow upper edge, “keel‐like” with denticles that continue along the antero‐outer edge, flattened ventral surface with a row of tubercles on the inner edge; in ♀, both P1 similar, less developed, narrower (Figure 10B) than that of the male, with denticulate dorsal edge, basal longitudinal row of tubercles of palm of propodus slightly marked, with a row of setae extending to the distal third of the fixed finger; fingers long, both of equal length, with corneous distal apex extending as an internal flange along the distal third, with small teeth on the remaining part of both inner edges.

P3 > P4 > P2 > P5. Legs 3 and 4 are approximately 1.5 times longer than maximum CW. P2: (Figure 10C) merus, dorsal outer face with (denticulated) serrated edge and numerous short setae, ventral outer (posterior) edge with teeth or tubercles sometimes hidden by a layer (occasionally dense) of short setae, covering the entire flattened ventral surface, inner border defined totally or partly by poorly developed tubercles (anterior ones more evident), outer and inner faces covered with smaller, more scattered setae, anterior border lobulated, length to height ratio is 2.4 ♀, 3.3 ♂; carpus smooth, with short setae, especially on the dorsal part; propodus smooth, with numerous setae, especially in dorsal and ventral areas; in the latter, other thicker and longer setae appear interspersed, scattered among shorter ones; length‐to‐height ratio is 2 for ♀, 2.6 for ♂; dactylus depressed, quite rectilinear in lateral view, triangular in dorsal or ventral view (Figure 10D), with a row of long setae on each side (laterals), another 2 rows on lateroventral, and other setae on the dorsal side, slightly shorter than propodus, dactylus to propodus length 1 ♀, 0.8 ♂; dactylus of ♀ slightly curved inwards.

P3 and P4: P3 (Figure 10E) slightly longer than P4 (Figures 11A, 12C), longer than maximum CW; morphologically similar to P2 except the inner ventral edge of the merus with tubercles on the anterior part and some on the posterior part (no obvious continuous row); propodus and carpus with numerous setae on the internal surface, with longer, thicker setae on the ventral and internal surfaces (especially in P4). Length‐to‐height ratios: P3 merus 2.4 for ♀, 3.2 for ♂, P4 merus 2.6 for ♀, 3.1 for ♂; P3 propodus 2.2 for ♀, 2.6 for ♂; P4 propodus 2.0 for ♀, 2.6 for ♂; P3 and P4 dactylus to propodus length 1.0 for ♀, 0.8 for ♂; dactylus as P2, lightly curved inwards (Figure 11Ca,b).

P5 (Figure 11B): with similar morphology as P2–P4 but smaller, without thick and long setae on carpus‐propodus; dactylus with dorsal edge somewhat concave, ventral edge convex (especially in proximal part); merus length‐to‐height ratio is 3.6 for ♂, propodus length‐to‐height ratio is 2.0 for ♂; dactylus to propodus length 1.2 for♂ (no data for females).

Thoracic sternum with anterior part (s1 and s2) resembling a truncated pyramid, with slightly concave lateral sides, apex concave and tuberculate (median tubercles more projected forward) (Figure 9D); with small tubercles at the level of thoracic sutures between sternites.

♂ and ♀ pleon (Figures 9D and 11C, 12D): with six distinct segments, plus telson; ♂ so6 with more or less straight lateral edges, tapering distally to meet broad, semicircular telson (Figure 9D).

G1: robust, with more or less uniform width along length, curved outwards, with distal area somewhat narrower, surrounded by a crown of setae (Figure 11E). G2: slender, small, G1/G2 ratio 5.0, pointed apex with a small distal lateral protuberance.

Vulva: oval‐shaped, large, with operculum, occupying the upper third of ♀ s6, with outer margin more or less rectilinear, inner surface gently inclined (Figure 11F).

*Maximum Sizes*: 7.25 mm of carapace width in males and 8.6 mm in females.

*Colouration*: All specimens were preserved in alcohol, so colour cannot be determined. However, the photos of two specimens of *A. atlanticus* deposited in BOLD, identified now as *S. mauritanicus* according to their COI sequences in BOLD, allow us to know about the colour of this species: beige carapace with small dark brown spots symmetrically distributed: one round and distinct spot in the centre of the mesogastric region, 2 and 4 less noticeable in the epigastric region, 2 distinct ones in the cardiac region along with other less noticeable ones, and irregular spots in the hepatic and branchial regions. Pereiopods without brown spots. The COI sequences of these two specimens fit 99.7% and 99.4% with the COI sequence PQ247038 of the male holotype of *S. mauritanicus* . These specimens are deposited in the University of Bergen, Norway, Natural History collections: 1♀, ZMBN92602, from Guinea‐Bissau, 99 m, 2012‐05‐20, R/V Dr. Fridtjof Nansen coll. (https://v4.boldsystems.org/index.php/Public_RecordView?processid=MIWAD193‐13) and 1 ♀, ZMBN92675, from Sahara, 28 m, 2012‐06‐13, R/V Dr. Fridtjof Nansen coll. (https://v4.boldsystems.org/index.php/Public_RecordView?processid=MIWAD266‐13).

*Habitat*: Sticky grey mud with some sand and shell gravel, spider crabs (*Inachus* sp., *Macropodia* sp.), bivalves, gastropods, tubeworms, hermit crabs and flatfish.

*Depth Range*: 28–99 m.

*Distribution*: Atlantic Ocean: from South of Western Sahara to Guinea‐Bissau (BOLD specimens).

*Remarks*: This African species has been misidentified with *D. atlanticus* , but both can be easily separated. This new species shows: a carapace less wide than that of *D. atlanticus* (CW/CL: 1.35–1.5 vs. 1.6–1.9), with the posterolateral borders diverging posteriorly less than in *D. atlanticus* ; external orbital cavity not well‐defined (delimited in *D. atlanticus* ); oral cavity with uniformly curved anterior edge (the antero‐central part more curved‐projected in *D. atlanticus* ); endostome with a central ridge (not in *D. atlanticus* ); pterygostomian region with tubercles in the frontal anterior part (not in *D. atlanticus* ), merus of third maxilliped without a well developed rounded basal projection (outwards perpendicularly) (it is too obvious in *D. atlanticus* ); the ischio‐merus joint of third maxilliped is slightly inclined, similar to that of *D. atlanticus* ; sternum without longitudinal sulcus on the anterior segments, also absent in *D. atlanticus* (it exists in *Gopkittisak gallardoi*); antennal flagellum extends well beyond the distal part of the cornea (reaches o slightly overreaches it in *D. atlanticus* ); walking leg: propodus P3, P4, clearly elongated compared to those of *D. atlanticus* in adults (length‐to‐height ratio is 2.0–2.6 vs. 1.15–1.4) and slightly longer than dactylus (dactylus‐to‐propodus length 0.8–1.2 vs. 0.8–1.1); dactylus too depressed, with a row of long setae on each side and another two lateroventral and dorsal, while in *D. atlanticus* it is less depressed (higher) with one lateral longitudinal rounded keel and two others on the dorsal and ventral sides.

Differences with the species of the genus *Asthenognathus* are mentioned in remarks of the genus *Schubartus* n. gen.

On the other hand, *S. mauritanicus* n. sp. is morphologically very close to *Schubartus ngankeeae* n. sp. (described below); its differences are discussed in the remarks of this latter species.

**FIGURE 13:**
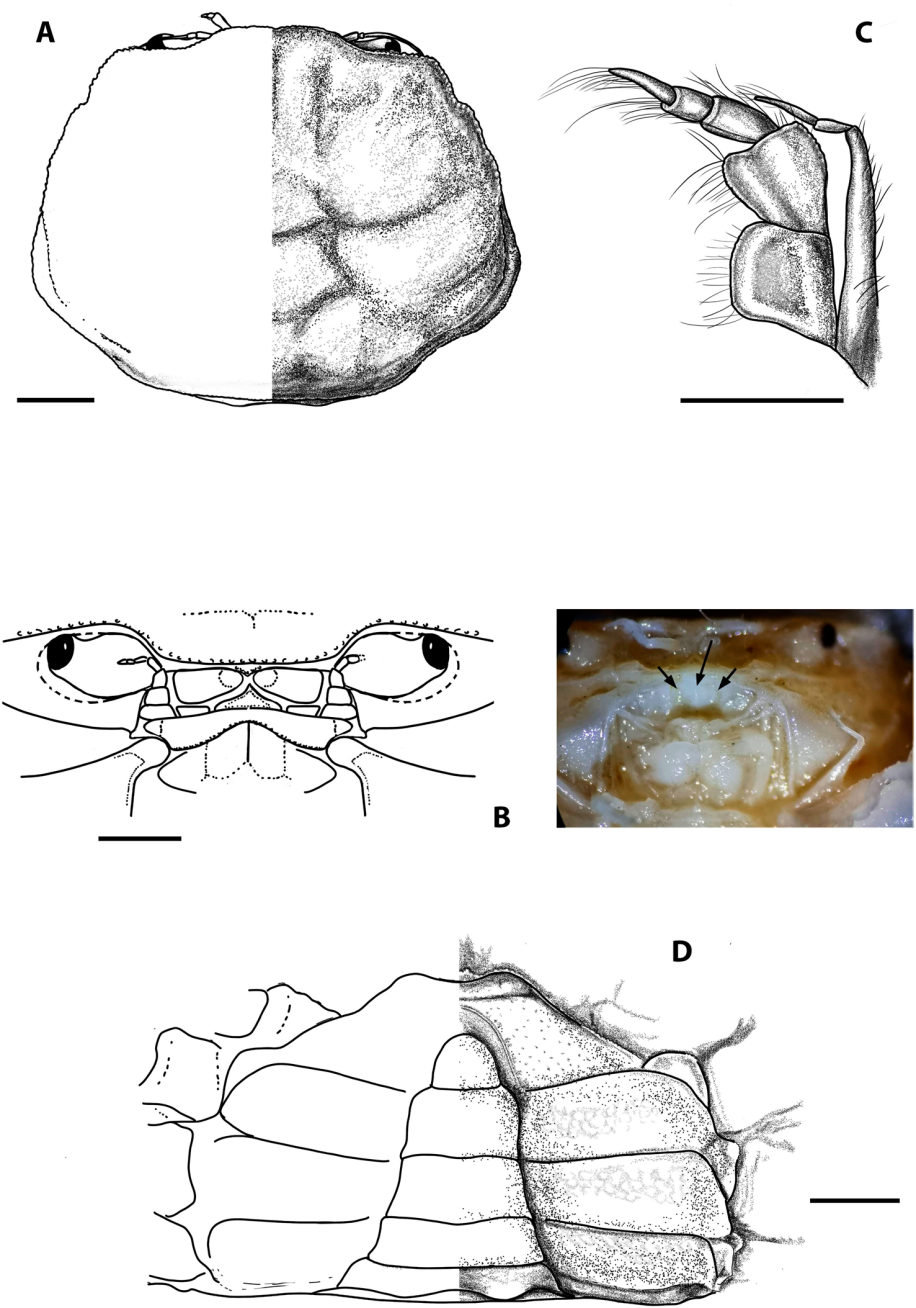
*Schubartus ngankeeae* n. gen., n. sp. (A) Carapace, dorsal view; (B) Partial fronto‐ventral view of carapace, buccal frame and endostome; (C) Left third maxilliped, ventral view; (D) Anterior part of sternum and male pleon, ventral view. (A–D) holotype (IEOCD‐CCLME12/1639‐1), 7.2 mm CW; (B) (photo), paratype IEOCD‐CCLME12/1639‐2), 7.24 mm CW, both from Guinea‐Bissau. Setae only indicated on some appendages. Scales 1 mm.

**FIGURE 14:**
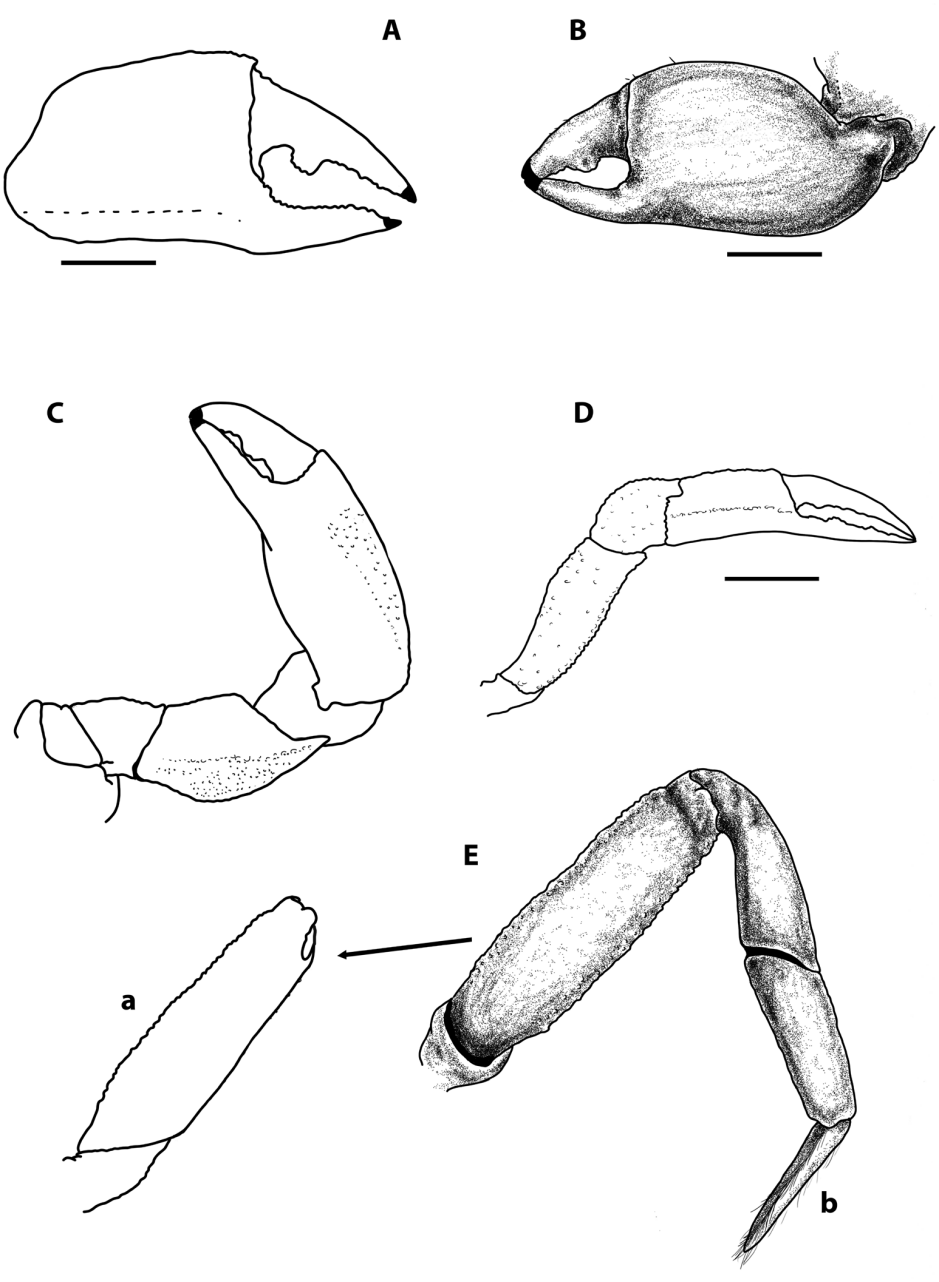
*Schubartus ngankeeae* n. gen., n. sp. (A) Right cheliped, male, outer view; (B) Left cheliped, male, outer view; (C) Left cheliped, male, ventral view; (D) Right cheliped, female, outer view; (E) P2 right, male, outer view, (a) detail of right merus inner view, (b) dactylus of P2, ventral view. (A, C, E) Male holotype (IEOCD‐CCLME12/1639‐1), 7.2 mm CW; (B) Male paratype (IEOCD‐CCLME12/1639‐2), 7.24 mm CW, both from Guinea‐Bissau. (D) Female (RMNH.CRUS.D40005) from Mauritania. Setae only indicated on some appendages. Scales 1 mm.

**FIGURE 15:**
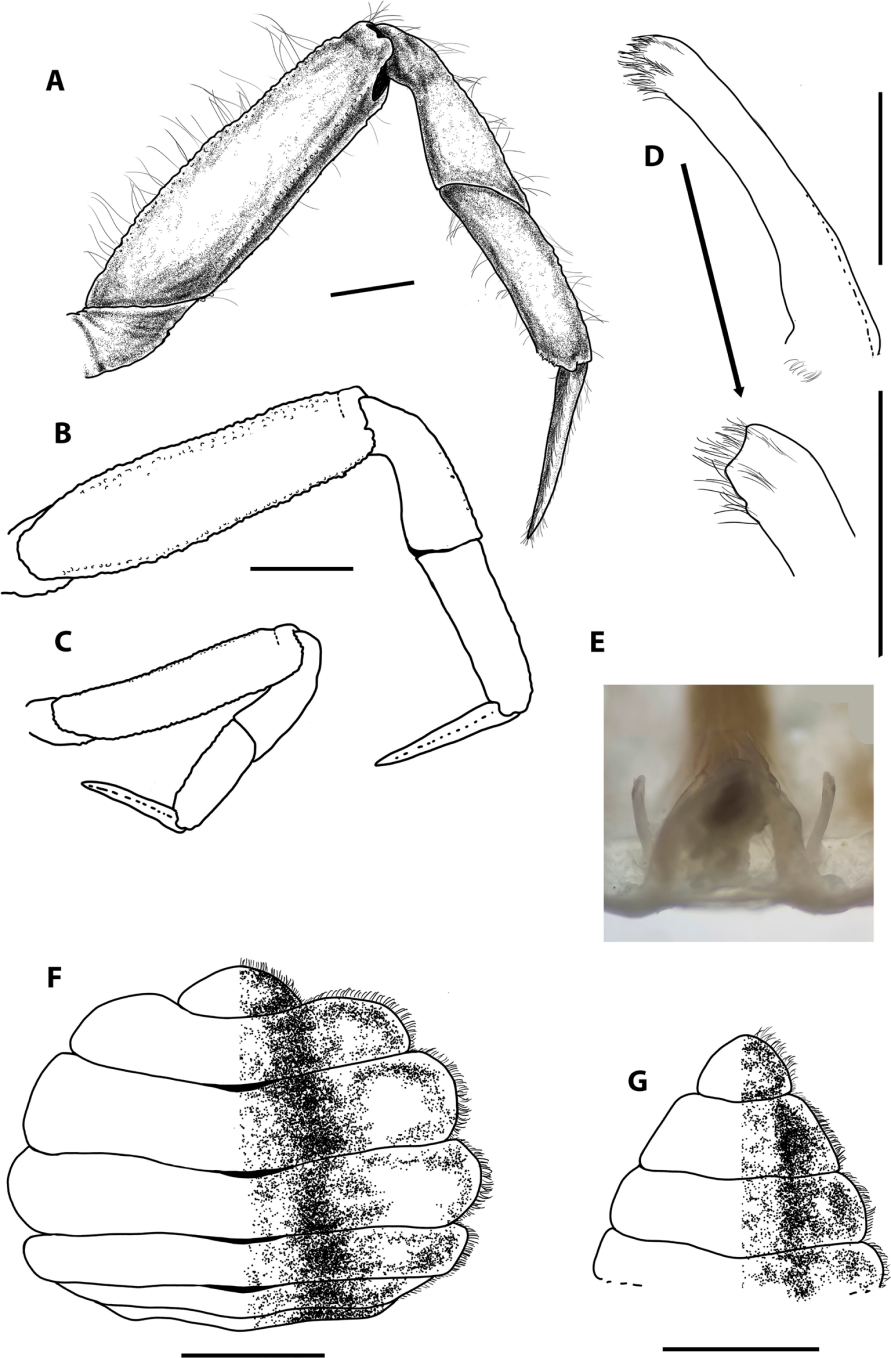
*Schubartus ngankeeae* n. gen., n. sp. (A) P3 right, male, outer view; (B) P4 right, male, outer view; (C) P5 right, male, outer view; (D) Right first pleopod of male, ventral view and detail of distal part; (E) G2, ventral view; (F) Pleon of adult female; (G) Pleon of immature female. (A, B, C) Male paratype (IEOCD‐CCLME12/1639‐2), 7.24 mm CW; (D, E) Male holotype (IEOCD‐CCLME12/1639‐1), 7.2 mm CW, both from Guinea‐Bissau; F: Adult female (RMNH.CRUS.D40008‐3), 6.2 mm CW, from Mauritania; G: Immature female (RMNH.CRUS.D40005), 6.1 mm CW, from Mauritania. Setae only indicated on some appendages. Scales 1 mm.

**FIGURE 16:**
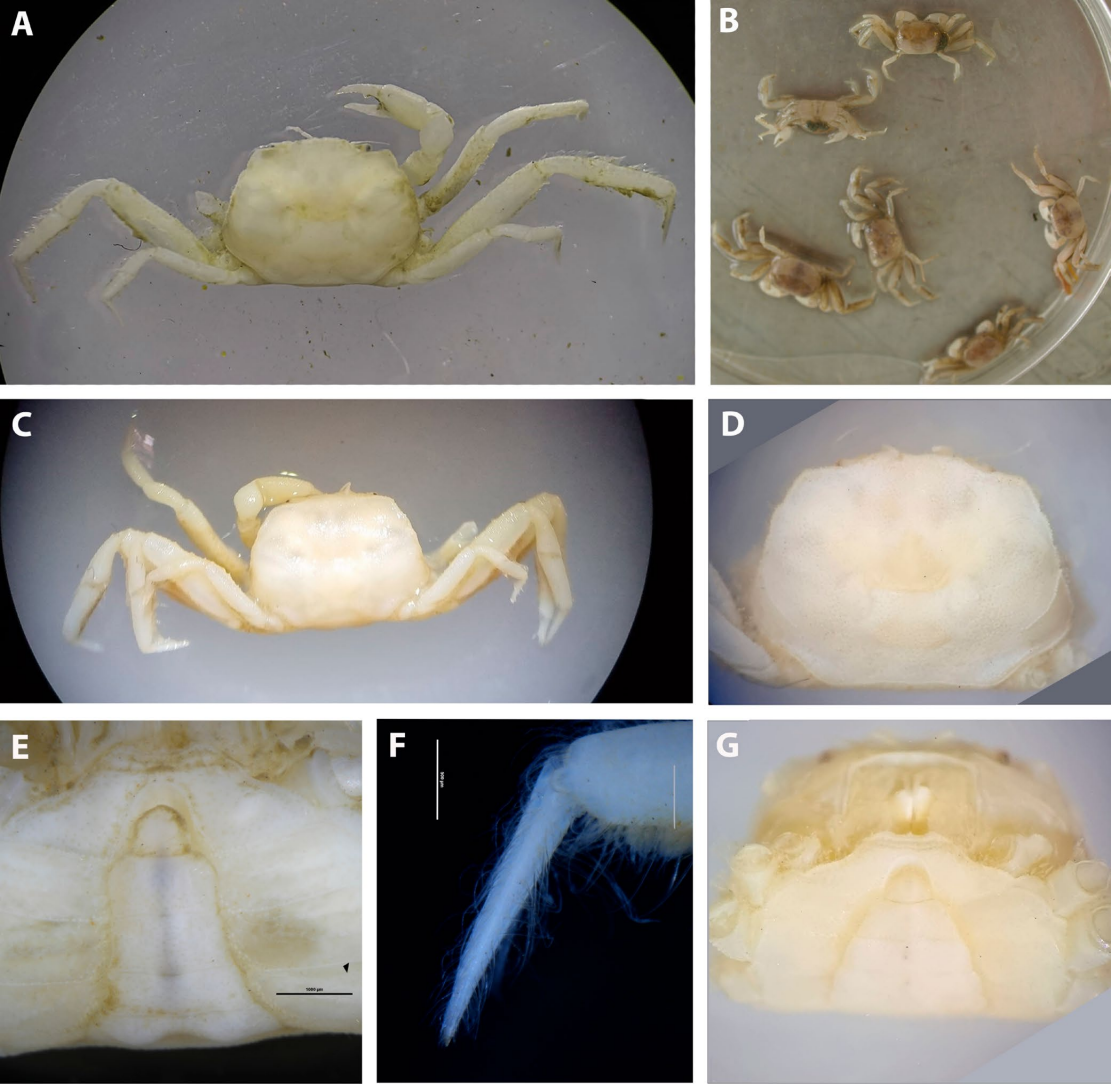
Photos of *Schubartus ngankeeae* n. gen., n. sp. (A) Male, holotype (IEOCD‐CCLME12/1639‐1), 7.2 mm CW, dorsal view; (B) Photo in live of the six specimens collected in 2012, but only two conserved; (C) Male, paratype (IEOCD‐CCLME12/1639‐2), 7.2 mm CW, dorsal view; (D) Carapace of female (RMNH.CRUS.D40005), 6.1 mm CW, dorsal view; (E) Pleon of the male, holotype; (F) P4 left dactylus of the male, paratype; (G) Pleon of immature female (RMNH.CRUS.D40005). (A–C, E–F) From Guinea‐Bissau; (D, G) From Mauritania.

***Schubartus ngankeeae*** n. gen., n. sp.

(Figures 8B, 13, 14, 15, 16)

(urn:lsid:zoobank.org:act:16A16020‐F816‐47FF‐ABDB‐700FA1DE7288)

**EXAMINED MATERIAL**. **HOLOTYPE**. GUINEA‐BISSAU • 1 ♂ (CW × CL: 7.2 × 5.21 mm), IEOCD‐CCLME12/1639‐1 “CCLME12” Expedition, Stn. 56, 20‐05‐2012, 11.9418 N, 17.2047 W, 74 m. Caught together with *Pseudomyra mbizi* Capart, 1951, sea stars, *Inachus* sp., *Parapenaeus longirostris* (Lucas, 1846), bivalves, gastropods, scaphopods and opistobranchia. Trawl net.

**PARATYPE**. GUINEA‐BISSAU • 1 ♂ (CW × CL: 7.24 × 5.21 mm), IEOCD‐CCLME12/1639‐2, same data as preceding.

**Other Specimens Examined**

**MAURITANIA** • 1 ♀ (6.2 × 4.7 mm), immature. RMNH.CRUS.D40005 (as *Asthenognathus atlanticus* Monod, 1933, det. C.H.J.M. Fransen, 1991), Stn. MAU.079 Mauritania, off Banc d'Arguin, 20°01′N, 17°23′W, 53 m, 14‐06‐1988. Dark‐grey muddy fine sand, tubeworms, ophiurids, *Tellina*, Van Veen grab (4×). • 1 ♀ (6.2 × 4.2 mm). RMNH.CRUS.D40008‐3 (as *Asthenognathus atlanticus* Monod, 1933, det. C.H.J.M. Fransen, 1991) “TYRO” Mauritania‐II Expedition 1988, Stn. MAU.101 Mauritania, off Banc d'Arguin, 19°43′N, 16°59′W, 61–72 m, 16‐06‐1988. Sticky grey mud with some sand and shell gravel, spider crabs (*Inachus* sp., *Macropodia* sp.), bivalves, gastropods, tubeworms, hermit crabs, flatfish. 2.4 m Agassiz trawl. • 1 ♂ (9.8 × 7.2 mm). RMNH.CRUS.D39701 (as *Asthenognathus atlanticus* Monod, 1933, det. C.H.J.M. Fransen, 1991) “CANCAP III” Expedition, Stn. 3155, Off Mauritania, 19°22′N, 16°51′W. 100–300 m, 31‐10‐1978. Sandy clay with shell gravel. Van Veen grab.

*Diagnosis*: As for the genus, in this species the carapace regions are more pronounced, with an evident transverse cervical groove and anterolateral margins with a well‐marked projection, another additional, less obvious in the epibranchial region. P3 and P4 are the longest, with merus, carpus and propodus relatively narrower; merus length‐to‐height ratios (P2 to P4) are 3.6–3.8 for ♂, 2.6–3.5 for ♀. Vulva oval‐shaped, large, with the outer margin slightly inclined.

Carapace broadly trapeziform, wider than long, < 1.5 times; anterolateral margins entire, with sinuosity at this level; posterolateral margin shorter than anterolateral margin; carapace regions more pronounced, with an evident transverse cervical groove; anterolateral margins with a well‐marked projection, another additional, less obvious in the epibranchial region; with an oblique granular ridge on the dorsal posterolateral part of the carapace; broad front, slightly bilobed with a median depression, gently flexed downward. Distal part of cornea resting outside the orbital cavity, on a long transverse orbital depression that extends latero‐ventrally to the lateral zone. Orbital area ventrally delimited by infraorbital ridge. Antennal flagellum relatively long, extending beyond distal part of cornea (approximately twice the length of ocular peduncles). Epistome triangular, short. Triangular pterygostomial region, with tubercles on the anterofrontal area. Endostome with longitudinal central ridge or projection. Third maxillipeds with wide separation between them when closed. P3 and P4 are the longest, with the merus, carpus, and propodus relatively narrower; merus length‐to‐height ratios (P2 to P4) are 3.6–3.8 for ♂, 2.6–3.5 for ♀; P5 is very reduced. Male pleon in pagoda. G1 robust, with uniform width along length, curved outward, with distal area surrounded by a crown of setae. Vulva oval‐shaped, large, with outer margin slightly inclined.

*Etymology*: The name refers to Dr. Ngan Kee Ng, a dedicated specialist in varunids, who made important contributions to the systematics of this group and unfortunately passed away in 2022.

*Description*: Carapace (Figures 13A, 16D): trapezoidal, wider than long, CW/CL 1.38–1.39 for ♂♂ (holotype‐paratype), 1.30–1.38 for ♀♀; maximum carapace width towards posterior part (third quarter); dorsal carapace finely grained, with tuberculated edges; anterolateral border with pronounced grainy ridge, especially in upper part, tuberculation less noticeable along posterolateral edge; bilobed front, rounded, sparsely projected forward; external orbital angles well‐defined, forming an almost straight angle with anterolateral edge, softening as size increases and, in some ♀♀, with smooth, rounded junction between both; anterolateral margin slightly sinuous, with a well‐marked projection or hepatic lobe, another less noticeable in the epibranchial region, also softening as size increases; posterolateral margin shorter than anterolateral margin, convex in first half, concave in second one, reaching posterior margin of carapace with curve angle; posterior border almost straight or slightly convex, with fine grainy ridge; approximately two times frontal border width; faintly marked carapace regions, with two slightly defined frontal lobes (with median depression), triangular gastric lobe, hepatic and intestinal regions poorly defined, rounded cardiac region, the most prominent; well‐defined cervical groove, slightly marked branchio‐cardiac grooves, granular keels or carinae, running backwards, in lateral branchial regions. Eyestalks moderately long, less than twice as long as wide, pronounced dilatation at base, particularly on ventral part, corneal area distally narrower; rounded cornea overlaps orbital cavity, hiding outer edge, resting on long transverse orbital depression (Figure 13B), extending latero‐ventrally to edge of anterolateral boundary. Orbital cavity open internally, so that eyestalks are in contact with basal segment of antenna; orbital area ventrally delimited by ridge running laterally upwards. Antennal flagellum extends well beyond distal part of cornea, reaching anterolateral edge. Antennular pits wider than long, antennules folding transversely, inner‐antennular septum very narrow. Suborbital region delimited by transverse, rectilinear, outwardly directed ventral suture. Epistome triangular, short, outer margins bordered by a tuberculated ridge. Triangular pterygostomial region, small tubercles on anterior area. Buccal cavity with anterior edge presenting a very open curve, meeting with nearly straight lateral edges. Endostome with a longitudinal central ridge and two inconspicuous latero‐longitudinal ridges.

Third maxillipeds (Figure 13C): with wide separation between them when closed; quadrangular ischium, except in the lower right corner where it is extended, convex inner margin with long setae, outer margin slightly concave, tuberculated and without setae, longitudinal groove in the center of this segment in semi‐ovoid shape; merus as long as wide, distally widened, outer margin with granulated edge, upper half slightly convex, proximal half more or less straight, without setae, convex inner margin with granulated margin, with long setae, longitudinal groove in the center of the segment, rounded upper angles, outer slightly more projected and denticulated; ischium—merus joint almost straight; palp: carpus > dactylus > propodus, with scarcely small setae; dactylus finger‐shaped, tapering at apex and with long setae at the distal part; distal part of exopod (without flagellum) reaches the distal quarter of merus, without exceeding it.

P1, right and left similar in males, well developed, globose (Figure 14A–C); outer face of propodus convex, smooth or with fine granulation, with some short setae, with longitudinal row of tubercles in basal quarter, extending from basal part of palm without reaching base of fixed finger, with setae extending through both fingers, dorsal edge curved, convex, with some little tubercles, not very marked, smooth and convex inner face; postero‐ventral‐internal area, at the joint with carpus, with tubercle with three obvious granules; palm of propodus with proximal part lower than distal one, dorsal edge curves downward, in posterior quarter, dorsal side of propodus convex, junction with carpus diagonal, ventral side almost rectilinear (slightly concave distally) and less globose than dorsal side; palm of propodus length‐to‐height ratio (measured medially) is 1.3 times longer than high; fingers of similar length, shorter than palm; dactylus to palm ratio (in mediun part) 1.2–1.7, with a wide space between them; fixed finger curved distally upwards, with some distal tubercles on inner edge, ending in corneous claw; dactylus, with dorsal edge very slightly curved, almost straight; ventrally with strong tooth in proximal first third (with 3 or 4 distal granules), followed by 4–5 small teeth reaching unarmed distal corneous tip; fixed finger with 9–11 small rounded teeth running along entire inner edge, tip corneous, unarmed; carpus convex, smooth or finely granulated, antero‐ventral edge tuberculate; merus triangular in section, convex external face, distal part higher than proximal, with dorsal area directed backwards and downwards, with narrow upper edge, “like keeled”, with denticles along antero‐outer edge, flattened ventral surface with row of tubercles on inner and outer edges. In ♀, both chelipeds are similar, less developed and narrower (Figure 14D) than that of the male, with a denticulate dorsal edge, a basal longitudinal row of tubercles of the palm slightly marked and a row of setae extending to the distal third of the fixed finger; fingers are too long and pointed, of equal length, longer than the palm, with a corneous distal apex extending as an internal flange along the distal third, with small teeth on the remaining part of both inner edges, bigger on the upper side.

Walking legs: elongated, slender, flattened laterally; P3 > P4 > P2 > P5; P2–P4 larger than the medium width of the carapace (P3 1.6 times longer than maximum CW, P4 1.5 times), P5 similar to CW.

P2 (Figure 14E): elongated and thin; merus (Figure 14E, Ea) dorsal outer face with (denticulated) serrated edge with abundant short setae, ventral outer edge with teeth or tubercles sometimes hidden by layer (sometime dense) of short setae and with some long ones in between, flattened ventral surface also covering, inner border of ventral face defined totally or partly by tubercles less developed (anterior ones more evident) and with some short setae; outer and inner faces of merus covered with smaller and more scattered setae, anterior border of merus lobulated, length‐(middle part)‐to‐height ratio is 3.6 for ♂♂ (types) (2.6–3.0 for ♀♀); carpus grained, longer than merus, without noticeable tuberculated edge, without setae; propodus grained, with smooth dorsal and ventral edges, setae on both sides, longer and more abundant on dorsal side, length‐to‐height ratio of 3.1 for ♂♂ (types), 2.2 for ♀♀; dactylus more or less triangular in shape, depressed, with longitudinal row of setae on lateral sides (external and internal) running from base to apex, dorsal and ventral sides with 2 similar longitudinal rows of setae, which delimit a ventral longitudinal central depression (Figure 14Eb), dactylus slightly shorter than propodus 0.9 ♂♂(types), 1.0 ♀♀.

P3 (Figure 15A): elongate and thin, slightly longer than P4; ischium denticulated; merus triangular in cross‐section, rectangular in lateral view with straight sides, anterior height greater than distal height, with subdistal dorsal cleft grained, granulated dorsal edge with setae, ventral edge also granulated with abundant setae, distal edge denticulated; length‐to‐height ratio of 3.6–3.8 for ♂♂ (types), 2.9–3.0 for ♀♀; carpus grained, dorsal edge granulated with scarce setae, ventral unarmed; propodus laterally flattened, grained, tuberculated dorsal edge, smooth ventral one, with long setae; length‐to‐height ratios 3.4–3.7 for ♂♂ (types), 2.6–2.8 for ♀♀; dactylus as P2, elongated, pointed, with triangle shape in dorsal view, with long setae row on lateral sides (external and internal), running from base to apex, dorsal and ventral sides with 2 similar longitudinal rows of setae, which delimit ventral longitudinal central depression; dactylus to propodus length ratio 0.7 for ♂♂ (types), 0.8 for ♀♀; dactylus P3 of ♀ slightly curved inwards.

P4 (Figure 15B): elongated and thin; ischium denticulated; merus triangular in cross‐section, rectangular in lateral view with straight sides, with subdistal dorsal cleft, dorsal edge grained, granulated with setae, ventral edge also granulated with abundant setae, distal edge denticulated, length‐to‐height ratios 3.6–3.8 for ♂♂ (types), 3.0–3.5 for ♀♀; carpus grained, dorsal edge granulated with setae, ventral unarmed; propodus laterally flattened, grained, dorsal and ventral edges almost smooth, dorsal with long setae, length‐to‐height ratios 3.0 for ♂♂ (types), 2.5–2.8 for ♀♀; dactylus elongated and pointed, with a triangle shape in dorsal view, with a long setae row on the lateral sides (external and internal) running from the base to apex, dorsal and ventral sides with 2 similar longitudinal rows of setae, which delimit a ventral longitudinal central depression; dactylus to propodus length 0.7 for ♂♂(types), 0.8 for ♀♀.

P5 (Figure 15C): reduced, slim, flattened laterally; ischium denticulated; merus finely grained with rectilinear and tuberculated edges, dorsal border with long setae, ventral scarcely, slightly tuberculated distally, length‐to‐height ratios 4.0–4.5 for ♂♂ (types), 3.9 for ♀♀; carpus grained, without tubercles, with long setae dorsally, less ventrally; propodus grained, without tubercles, with long setae ventrally, length‐to‐height ratios 2.2–2.4 for ♂♂ (types) (2.0 for ♀♀); dactylus elongated, pointed, with triangle shape in dorsal view, with a long setae row on lateral sides (external and internal), running from the base to apex; dactylus to propodus length ratios 0.9 for ♂♂ (types), 1.1 ♀♀.

Rounded sternum (Figure 13D), anterior section as a truncated pyramid with slightly concave lateral sides, a concave and a tuberculate apex (with bigger granules). Thoracic sternites s4–7 with granules in the upper half, tuberculated sutures between sternites (Figure 13D).

G1 (Figure 15D): thick, with more or less uniform width along the entire length, curved outward, without setae along; the distal part truncates and is rectangular, somewhat narrower and surrounded by a crown of setae. G2 is slender and small, with a ratio of G1/G2 6.0, narrower with a blunt apex, with a small lateral protuberance (Figure 15E).

Pleon ♂ (Figures 13D and 16D) and ♀ (Figures 15F,G and 16G): with six distinct segments, plus telson. In adult ♂, so6 with more or less straight lateral edges. Rounded telson, narrower than so6, so distal lateral ends of this segment protrude.

Vulva: oval‐shaped, large, with operculum, occupying upper third of 6th female sternum, with outer margin slightly inclined inside (anterior) to outside (posterior).

*Maximum Sizes*: 9.8 mm CW in males and 6.5 mm in females.

*Colouration*: Based on the specimen types: The carapace is distinctly divided into two colour zones: light brown from the cervical groove forward and beige from the cervical groove backward, with a central, regular darker stripe covering the cardiac and intestinal regions. The chelipeds and walking legs are pale, and the ventral side is also pale beige.

*Habitat*. Soft bottom, caught together *Pseudomyra mbizi*, sea stars, *Inachus* sp., *Parapenaeus longirostris* , bivalves, gastropods, scaphopods and opistobranchia.

*Depth Range*: 53 to 100–300 m.

*Distribution*: Currently only known from Mauritania and Guinea‐Bissau.

*Remarks*: *S. ngankeeae* is morphologically very close to *S. mauritanicus* . The most noticeable differences are the more elongated and thinner walking legs in ♂♂ of *S. ngankeeae*. As a summary, the length‐toto‐height ratios of the merus of P2–P4 are for ♂♂: less than 3.6 in *S. mauritanicus* vs. more than 3.6 in *S. ngankeeae*, and for females: less than 2.6 in *S. mauritanicus* vs. more than 2.6 in *S. ngankeeae*. However, in a large male specimen of *S. ngankeeae* (9.8 mm CW), these ratios are 2.4 (P2) and 3.1 (P3); this could indicate that the walking legs of males show an allometric growth with size.

Most significant differences are found in the length‐to‐height ratios of the propodus of ambulatory legs P2 to P4, which in *S. ngankeeae* are considerably longer than in *S. mauritanicus* ; these ratios are for *S. ngankeeae* P2 3.1 for ♂♂, 2.2 for ♀♀; P3 3.4–3.7 for ♂♂, 2.6–2.8 for ♀♀ and P4 3.0 for ♂♂, 2.5–2.8 for ♀♀ and for *S. mauritanicus* P2 2.6 for ♂♂, 2.0 for ♀♀, P3 2.6 for ♂♂, 2.2 for ♀♀ and P4 2.6 for ♂♂, 2.0 for ♀♀.

Differences have also been found in the CW to CL ratio between the two species; for *S. mauritanicus*, 1.34 for ♂♂, 1.5 for ♀♀ vs. *S. ngankeeae*, 1.36–1.39 for ♂♂, 1.3–1.38 for ♀♀. The females of *S. mauritanicus* seem to be wider than those of *S. ngankeeae*. Other slight differences are found in the carapace: *S. ngankeeae* shows the carapace regions a little more pronounced, with the external orbital angles slightly more marked, and the anterolateral margins with a well‐marked hepatic lobe, and another less noticeable in the epibranchial region (less marked in some big specimens), not clear in the specimens of *S. mauritanicus* .

The molecular differences between both species justify their separation, but morphologically they are very similar with few differences, mainly based on morphometric data obtained from a few specimens of different sizes and sexes; therefore, these values should be confirmed with more specimens.

## 4 Discussion

### 4.1 Taxonomic Remarks, Biogeography and Phylogenetic Analyses

This group of species of the genera *Dudekemus* n. gen. and *Schubartus* n. gen. shows an East Atlantic distribution (the first also in the Mediterranean Sea). *Dudekemus atlanticus* n. comb. occurs from Northern Europe to Morocco; Western Sahara is included within the “Temperate Northern Atlantic: Northern European Seas and Lusitanian, including the Mediterranean Sea” marine ecoregion, while the other two species, belonging to the genus *Schubartus* n. gen., somewhat more southerly, are found in the “Tropical Atlantic ecoregion: West African Transition (Sahelian upwelling) and Gulf of Guinea” (Spalding et al. 2007). These African marine geographic areas are within the influence of the Canarian Current Upwelling System (Gómez‐Letona et al. 2017), which is related to relatively cold and nutrient‐rich waters, driving the vertical cell of coastal upwelling that makes it one of the most productive marine regions; so, the upwelling intensity‐driven abundance patterns of some species, such as the larval fish (Tiedemann et al. 2017), and where several marine protected areas (MPAs) are located (Failler et al. 2020) that need continuous and effective management, despite insufficient human and institutional capacities and lack of economic resources (Guénette et al. 2014). However, there are considerable uncertainties about its full distribution range (due to its size and presumed habitats, which make it difficult to collect), which could be much wider than currently known.

The coexistence of the two *Schubartus* species ( *S. mauritanicus* n. gen., n. sp. and *S. ngankeeae* n. gen., n. sp.) in the South Western Sahara–Mauritania and the presence of *D. atlanticus* n. gen., n. comb. in the Western Sahara could be related to the fact that this sector (Western Sahara‐Mauritania) is a boundary between biogeographical ecoregions, where species coexist, increasing biodiversity. Thus, the richness and the presence of endemic species justify its consideration as a biodiversity hotspot (Halpern et al. 2015; Costello et al. 2022), despite the fact that the information on the existing faunal richness in this area is limited compared to others, such as the Mediterranean (Coll et al. 2010), and most of the data come from general reviews, scientific expeditions and fishing campaigns (Monod 1956; Anadón 1981; Manning and Holthuis 1981; Fransen 1991; De Matos‐Pita et al. 2017), with the coastal and rocky areas being little studied.

On the other hand, it is worth mentioning that Guinot and Ribeiro (1962) cited specimens of *Asthenognathus atlanticus* in Angola. However, without figures and/or morphological comments and based on the available information, this record must be attributed to *Schubartus*, but we cannot assign them to one of the two species without seeing the specimens and analyzing their DNA; however, due to the CW/CL ratio, some of them could perhaps be *S. mauritanicus* .

Regarding new species, it is worth mentioning that the existence of cryptic or pseudocryptic species is increasing with the development of molecular taxonomy, associated with morphology, “integrative taxonomy” Thus, new species have recently been described in Eastern Atlantic waters, whose morphological variability was not known to be due to geographic variability or if they were simply different but morphologically very close species. This is the case of some *Pinnotheres* Bosc, 1801 (Cuesta et al. 2019); *Inachus* Weber, 1795 (García‐Raso et al. 2022), *Polybius* Leach, 1820 (García‐Raso et al. 2024) and the clarification of *Diogenes* Dana, 1851 spp. from the Eastern Atlantic (Almón et al. 2021, 2022), among others.

The great morphological similarity between both *Schubartus* spp., with overlapping ranges in the eastern Atlantic and the exploitation of similar ecological niches, such as soft bottoms, likely associated with Polychaeta or another burrowing group (as in *Dudekemus atlanticus* n. gen., n. comb.), suggests that they represent recently diverged sister species. These species may have been selected for high levels of behavioral incompatibility and reproductive isolation as a result of transient allopatry followed by secondary contact (as has been proposed for some sibling snapping shrimp species by Mathews et al. 2002). Molecular data are also essential to identify anomalous specimens, juveniles with poorly defined characters, and especially among pseudocryptic species with a very close morphology that differs with size and sex (this is the case of some specimens of *Schubartus*, as the male RMNH. CRUS. D39701). In addition, molecular data have allowed us to clarify phylogenetic relationships, which is especially interesting with species that show morphological convergence and/or are very similar, with small changes that are difficult to assess by morphological analysis alone. In this regard, the present manuscript addresses the problem of the Varunidae, that after the interesting study by Ng (2006), where she established several new subfamilies, genera and species and replaced some taxa out of Varunidae, there are still several unresolved relationships at the internal familial level, as shown by a recent study using molecular techniques (Tsang et al. 2022) that considers that Varunidae is not monophyletic in its present composition. As an example, the case of *Hemiplax*, also mentioned in the introduction, is one whose taxonomic position is unclear, and perhaps it should have its own subfamily (according to Ng PKL, personal communication). Unfortunately, the analysis by Tsang et al. (2022) did not include members of the Asthenognathinae.

### 4.2 Updated Key to Asthenognathinae Stimpson, 1858 and Schubartinae n. Subf

A new taxonomic key for the species of the subfamilies Asthenognathinae and Schubartinae, with the description of the two new genera (*Dudekemus* n. gen. and *Schubartus* n. gen.) and the two new species ( *S. mauritanicus* n. gen., n. sp. and *S. ngankeeae* n. gen., n. sp.), is presented.

**Table:** 

1	Carapace wider than long, more than one and a half times. Length of the frontal region (anterior part) less than half the maximum width of the carapace. Lateral margins of the sixth abdominal segment convergent towards the distal part	*Asthenognathus* Stimpson, 1858 (from Southern China) 2
1′	Carapace wider than long, less than one and a half times. Length of the frontal region (anterior part) more than half the maximum width of carapace. Lateral margins of sixth abdominal segment with parallel or convergent lateral margins	*Dudekemus* n. gen. and *Schubartus* n. gen. (from Eastern Atlantic Ocean) 3
2	Posterior border of carapace one and a half times as long as front‐orbital width	*A. inaequipes* Stimpson, 1858
2′	Posterior border of carapace subequal to front‐orbital width	*A. hexagonus* Rathbun, 1909
3	Endostome without longitudinal central ridge. Lateral margins of sixth abdominal segment converge towards distal part. P3 and P4 with broadened merus, carpus and propodus (adults)	*Dudekemus atlanticus* (Monod, 1933) n. gen., n. comb
3′	Endostome with longitudinal central ridge. Lateral margins of sixth abdominal segment with parallel lateral margins. P3 and P4 with not broadened merus, carpus and propodus	*Schubartus* n. gen. 4
4	Slender walking legs, length/height ratio of merus P2–P4 more than 3.6 in males and more than 2.6 (in females). CW/CL for males 1.36–1.39, for females 1.30–1	*S. ngankeeae* n. gen., n. sp.
4′	Shorter walking legs, length/height ratio of merus P2–P4 less than 3.3 in males and less than 2.6 in females. CW/CL for males around 1.34, for females around 1.5	*S. mauritanicus* n. gen., n. sp.

## Author Contributions

**Isabel Muñoz:** conceptualization (equal), data curation (equal), formal analysis (lead), investigation (equal), methodology (equal), project administration (equal), resources (equal), validation (equal), visualization (equal), writing – original draft (lead), writing – review and editing (equal). **J. Enrique García‐Raso:** conceptualization (equal), data curation (equal), formal analysis (equal), funding acquisition (lead), investigation (equal), methodology (equal), project administration (equal), resources (lead), supervision (lead), validation (equal), visualization (equal), writing – original draft (equal), writing – review and editing (equal). **Jose A. Cuesta:** conceptualization (equal), data curation (equal), formal analysis (lead), funding acquisition (equal), investigation (equal), methodology (equal), project administration (equal), resources (equal), software (equal), supervision (lead), validation (equal), visualization (equal), writing – original draft (equal), writing – review and editing (equal).

## Conflicts of Interest

The authors declare no conflicts of interest.

## Data Availability

The data underlying this article are available in the article. DNA sequences and related data are publicly available on GenBank (https://www.ncbi.nlm.nih.gov/genbank/). The data associated with each of the specimens examined are included in the text, in the appropriate sections. The accession number for sequences downloaded from public databases is included in Table 2 along with those generated for this project.

## References

[ece371712-bib-0001] Ahyong, S. T. , and P. K. L. Ng . 2009. “Aphanodactylidae, a New Family of Thoracotreme Crabs (Crustacea: Brachyura) Symbiotic With Polychaete Worms.” Zootaxa 2289: 33–47. 10.11646/zootaxa.2289.1.3.

[ece371712-bib-0091] Akaike, H. 1998. “Information Theory and an Extension of the Maximum Likelihood Principle.” In Selected Papers of Hirotugu Akaike. Springer Series in Statistics, edited by E. Parzen , K. Tanabe , and G. Kitagawa . Springer. 10.1007/978-1-4612-1694-0_15.

[ece371712-bib-0002] Almón, B. , J. A. Cuesta , and J. E. García‐Raso . 2022. “Two New Hermit Crab Species of *Diogenes* (Crustacea: Decapoda: Diogenidae) From Atlanto‐Mediterranean Coasts of Iberian Peninsula: Poleward Migrants or Merely Overlooked Indigenous Species?” Ecology and Evolution 12: e8844. 10.1002/ece3.8844. 35600683 PMC9120568

[ece371712-bib-0003] Almón, B. , J. A. Cuesta , C. D. Schubart , L. Armenia , and J. E. García‐Raso . 2021. “Redescription of the Hermit Crab *Diogenes pugilator* (Decapoda: Anomura) Reveals the Existence of a Species Complex in the Atlanto‐Mediterranean Transition Zone, Resulting in the Resurrection of *D. Curvimanus* and the Description of a New Species.” Zoological Journal of the Linnean Society 195: 1116–1146. 10.1093/zoolinnean/zlab093.

[ece371712-bib-0004] Anadón, R. 1981. “Crustáceos Decápodos Recogidos Durante la Campaña “Atlor VII” en las Costas Noroccidentales de África (Noviembre 1975).” Resultados Expediciones Científicas 9: 151–159. http://hdl.handle.net/10261/156267.

[ece371712-bib-0005] Astrin, J. , and P. Stüben . 2008. “Phylogeny in Cryptic Weevils: Molecules, Morphology and New Genera of Western Palaearctic Cryptorhynchinae (Coleoptera: Curculionidae).” Invertebrate Systematics 22, no. 5: 503. 10.1071/is07057.

[ece371712-bib-0006] Bakker, H. , A. Gill , and J. Creuwels . 2024. Naturalis Biodiversity Center (NL) ‐ Crustacea. Naturalis Biodiversity Center.

[ece371712-bib-0007] Bocquet, C. 1963. “Remarques Morphologiques et Systématiques sur le Crabe *Tritodynamia atlantica* (Th. Monod) (=*Asthenognathus atlanticus* Th. Monod) Présent Dans la Région de Roscoff.” Cahiers de Biologie Marine 4: 65–79.

[ece371712-bib-0008] Bocquet, C. 1965. “Stades Larvaires et juvéniles de *Tritodynamia Atlantica* (Th. Monod) (=*Asthenognathus atlanticus* Th. Monod) et Position systématique de Ce Crabe.” Cahiers de Biologie Marine 6: 407–418.

[ece371712-bib-0009] Chagnoux, S. 2024. The Crustaceans Collection (IU) of the Muséum national d'Histoire Naturelle (MNHN ‐ Paris). Version 68.374. MNHN ‐ Museum National d'Histoire Naturelle.

[ece371712-bib-0010] Coll, M. , C. Piroddi , J. Steenbeek , et al. 2010. “The Biodiversity of the Mediterranean Sea: Estimates, Patterns, and Threats.” PLoS One 5, no. 8: e11842. 10.1371/journal.pone.0011842. 20689844 PMC2914016

[ece371712-bib-0011] Costello, M. J. , M. M. Vale , W. Kiessling , S. Maharaj , J. Price , and G. H. Talukdar . 2022. “Cross‐Chapter Paper 1: Biodiversity Hotspots.” In Climate Change 2022: Impacts, Adaptation and Vulnerability. Contribution of Working Group II to the Sixth Assessment Report of the Intergovernmental Panel on Climate Change, edited by H.‐O. Pörtner , D. C. Roberts , M. Tignor , et al., 2123–2161. Cambridge University Press. 10.1017/9781009325844.018.

[ece371712-bib-0012] Crandall, K. , and J. Fitzpatrick . 1996. “Crayfish Molecular Systematics: Using a Combination of Procedures to Estimate Phylogeny.” Systematic Biology 45, no. 1: 1–26. 10.1093/sysbio/45.1.1.

[ece371712-bib-0013] Cuesta, J. A. , J. E. García‐Raso , P. Abelló , E. Marco‐Herrero , L. Silva , and P. Drake . 2019. “A New Species of Pea Crab From South‐Western Europe (Crustacea, Decapoda, Brachyura): Species Description, Geographic Distribution and Population Structure With an Identification Key to European Pinnotheridae.” Journal of the Marine Biological Association of the United Kingdom 99: 1141–1152. 10.1017/S0025315419000018.

[ece371712-bib-0014] Cuesta, J. A. , C. D. Schubart , and D. L. Felder . 2005. Systematic Position of the Asthenognathidae Stimpson, 1858 and Pseudopinnixa carinata Ortman (Decapoda, Brachyura): New Findings From Larval and DNA Comparisons. Sixth International Crustacean Congress, 18–22 July 2005.

[ece371712-bib-0015] Cuesta, J. A. , C. D. Schubart , and G. Rodríguez . 2000. “Larval Development of *Brachynotus sexdentatus* Risso (Crustacea, Decapoda, Varunidae) Reared in the Laboratory, With Notes on Larval Characters of the Varunidae.” Invertebrate Reproduction and Development 38, no. 3: 207–223. 10.1080/07924259.2000.9652456.

[ece371712-bib-0016] Dai, A.‐Y. , and S.‐L. Yang . 1991. Crabs of the China Seas. China Ocean Press, Springer‐Verlag.

[ece371712-bib-0017] Darriba, D. , G. L. Taboada , R. Doallo , and D. Posada . 2012. “jModelTest 2: More Models, New Heuristics and Parallel Computing.” Nature Methods 9: 772. 10.1038/nmeth.2109 PMC4594756 22847109

[ece371712-bib-0018] Davie, P. J. F. , D. Guinot , and P. K. L. Ng . 2015a. “Systematics and Classification of Brachyura.” In Treatise on Zoology–Anatomy, Taxonomy, Biology – The Crustacea, Complementary to the Volumes Translated From the French of the Traité de Zoologie, edited by P. Castro , P. J. F. Davie , D. Guinot , F. R. Schram , and J. C. von Vaupel Klein , 1049–1130. Brill, Leiden.

[ece371712-bib-0019] Davie, P. J. F. , D. Guinot , and P. K. L. Ng . 2015b. “Anatomy and Functional Morphology of Brachyura.” In Treatise on Zoology – Anatomy, Taxonomy, Biology – The Crustacea, Complementary to the Volumes Translated From the French of the Traité de Zoologie, edited by P. Castro , P. J. F. Davie , D. Guinot , F. Schram , and C. von Vaupel Klein , vol. 9, 11–163. Brill, Leiden.

[ece371712-bib-0020] Davie, P. J. F. , and N. Ng . 2007. “Two New Subfamilies of Varunidae (Crustacea: Brachyura), with Description of Two New Genera.” Raffles Bulletin of Zoology 16: 257–272.

[ece371712-bib-0021] De Matos‐Pita, S. S. , S. Castillo , and F. Ramil . 2017. “Contribution to the Knowledge of the Deep Brachyuran Fauna (Crustacea: Decapoda) in Waters Off Mauritania (NW Africa).” Journal of the Marine Biological Association of the United Kingdom 97: 1273–1305. 10.1017/S002531541600062X.

[ece371712-bib-0022] DecaNet . 2024. “DecaNet.” https://www.decanet.info on 2024‐07‐25 [Dataset]. VLIZ.

[ece371712-bib-0023] d'Udekem d'Acoz, C. 1999. “Inventory and Distribution of the Decapod Crustaceans From the Northeastern Atlantic, the Mediterranean and the Adjacent Continental Waters North of 25°N.” In Collection Patrimoines Naturels, vol. 40, 2383. Muséum national d'Histoire naturelle.

[ece371712-bib-0024] Eibye‐Jacobsen, D. , L. Pavesi , T. Schiøtte , M. V. Sørensen , and J. Olesen . 2024. NHMD Invertebrate Zoology Collection. Natural History Museum of Denmark.

[ece371712-bib-0025] Estoup, A. , C. Largiader , E. Perrot , and D. Chourrout . 1996. “Rapid One‐Tube DNA Extraction for Reliable PCR Detection of Fish Polymorphic Markers and Transgenes.” Molecular Marine Biology and Biotechnology 5: 295–298.

[ece371712-bib-0026] Faasse, M. , H. Gheerardyn , R. Witbaard , and J. Cuperus . 2021. “First Record of the Rare Crab *Asthenognathus atlanticus* Monod, 1933 (Crustacea: Brachyura: Varunidae) in the North Sea.” International Journal of Oceanography and Hydrobiology 50, no. 3: 352–358. 10.2478/oandhs-2021-0030.

[ece371712-bib-0027] Failler, P. , G. Touron‐Gardic , B. Drakeford , O. Sadio , and M.‐S. Traoré . 2020. “Perception of Threats and Related Management Measures: The Case of 32 Marine Protected Areas in West Africa.” Marine Policy 117: 103936. 10.1016/j.marpol.2020.103936.

[ece371712-bib-0090] Folmer, O. , M. Black , W. Hoeh , R. Lutz , and R. Vrijenhoek . 1994. “DNA Primers for Amplification of Mitochondrial Cytochrome c Oxidase Subunit I from Diverse Metazoan Invertebrates.” Molecular Marine Biology and Biotechnology 3, no. 5: 294–299. 10.1051/kmae:2006008. 7881515

[ece371712-bib-0028] Fransen, C. H. J. M. 1991. Preliminary Report on Crustacea Collected in the Eastern Part of the North Atlantic During CANCAP and Mauritania Expeditions of the Former Rijksmuseum Van Natuurlijke Historie, Leiden, 200. National Natuurhistorich Museum.

[ece371712-bib-0029] García‐Raso, J. E. , C. d'Udekem d'Acoz , A. Moukrim , C. D. Schubart , and J. A. Cuesta . 2024. “A New Cryptic Species of Polybiidae (Crustacea: Decapoda: Portunoidea) From the East Atlantic, With Considerations on the Genus *Polybius* .” European Journal of Taxonomy 930: 277–313. 10.5852/ejt.2024.930.2501.

[ece371712-bib-0030] García‐Raso, J. E. , E. González‐Ortegón , F. Palero , and J. A. Cuesta . 2022. “A New Cryptic Species of *Inachus* Weber, 1795 (Decapoda: Brachyura: Inachidae) From European Waters and an Updated Identification Key to the Species of *Inachus* With Two Protogastric Tubercles.” Journal of Crustacean Biology 42: 1–13. 10.1093/jcbiol/ruac035.

[ece371712-bib-0031] Glémarec, M. , and C. Hily . 1979. “Nouvelles données sur la répartition de *Tritodynamea atlantica* (Balls, 1922).” Cahiers de Biologie Marine 20: 449–505.

[ece371712-bib-0032] Gómez‐Letona, M. , A. G. Ramos , J. Coca , and J. Arístegui . 2017. “Trends in Primary Production in the Canary Current Upwelling System—A Regional Perspective Comparing Remote Sensing Models.” Frontiers in Marine Science 4: 370. 10.3389/fmars.2017.00370.

[ece371712-bib-0033] Green, M. R. , and J. Sambrook . 2018. “Touchdown Polymerase Chain Reaction (PCR).” Cold Spring Harbor Protocols 5: 5133. 10.1101/pdb.prot095133 29717053

[ece371712-bib-0034] Guénette, S. , B. Meissa , and D. Gascuel . 2014. “Assessing the Contribution of Marine Protected Areas to the Trophic Functioning of Ecosystems: A Model for the Banc D'arguin and the Mauritanian Shelf.” PLoS One 9, no. 4: e94742. 10.1371/journal.pone.0094742. 24728033 PMC3984206

[ece371712-bib-0035] Guinot, D. , N. K. Ng , and P. A. Rodríguez Moreno . 2018. “Review of Grapsoid Families for the Establishment of a New Family for Leptograpsodes Montgomery, 1931, and a New Genus of Gecarcinidae H. Milne Edwards, 1837 (Crustacea, Decapoda, Brachyura, Grapsoidea MacLeay, 1838).” Zoosystema 40, no. 26: 547–604. 10.5252/zoosystema2018v40a26.

[ece371712-bib-0036] Guinot, D. , and A. Ribeiro . 1962. Sur une collection de Crustacés Brachyoures des iles du Cap‐Vert et de l'Angola. Memorias da Junta de Investigaçoes do Ultramar.

[ece371712-bib-0037] Halpern, B. S. , M. Frazier , J. Potapenko , et al. 2015. “Spatial and Temporal Changes in Cumulative Human Impacts on the World's Ocean.” Nature Communications 6: 7615. 10.1038/ncomms8615. PMC4510691 26172980

[ece371712-bib-0039] Huelsenbeck, J. P. , and B. Rannala . 2004. “Frequentist Properties of Bayesian Posterior Probabilities of Phylogenetic Trees Under Simple and Complex Substitution Models.” Systematic Biology 53: 904–913. 15764559 10.1080/10635150490522629

[ece371712-bib-0040] Ingle, R. W. 1980. British Crabs: 1–222. British Museum (Natural history), Oxford University Press.

[ece371712-bib-0041] Jiang, W. , H.‐L. Chen , and R.‐Y. Liu . 2007. “On Two New Record Species of the Genus *Asthenognathus* (Crustacea: Pinnotheridae) From the China Seas.” Oceanologia et Limnologia Sinica 38: 77–83.

[ece371712-bib-0042] Jourde, J. , S. Alizier , C. Dancie , et al. 2012. “First and Repeated Records of the Tropical Temperate Crab *Asthenognathus atlanticus* Monod, 1932 (Decapoda: Brachyura) in the Eastern Part of the Bay of Seine (Eastern English Channel, France).” Cahiers de Biologie Marine 53, no. 4: 525–532.

[ece371712-bib-0043] Katoh, K. , and D. M. Standley . 2013. “MAFFT Multiple Sequence Alignment Software Version 7: Improvements in Performance and Usability.” Molecular Biology and Evolution 30: 772–780. 23329690 10.1093/molbev/mst010 PMC3603318

[ece371712-bib-0044] Kearse, M. , R. Moir , A. Wilson , et al. 2012. “Geneious Basic: An Integrated and Extendable Desktop Software Platform for the Organization and Analysis of Sequence Data.” Bioinformatics 28: 1647–1649. 22543367 10.1093/bioinformatics/bts199 PMC3371832

[ece371712-bib-0045] Kitaura, J. , K. Wada , Y. Fukui , and C. L. Mclay . 2010. “Molecular Phylogenetic Position of the New Zealand Sentinel Crab, *Macrophthalmus* (*Hemiplax*) *hirtipes* (Jacquinot, in Hombron & Jacquinot, 1846) (Decapoda, Brachyura, Macrophthalmidae).” Crustaceana 83: 1315–1326.

[ece371712-bib-0046] Kitaura, J. , K. Wada , and M. Nishida . 2002. “Molecular Phylogeny of Grapsoid and Ocypodoid Crabs With Special Reference to the Genera *Metaplax* and *Macrophthalmus* .” Journal of Crustacean Biology 22: 682–693.

[ece371712-bib-0047] Ko, H. S. , and S. H. Lee . 2012. Invertebrate Fauna of Korea. Crabs and Zoeas I Arthropoda: Crustacea: Decapoda: Brachyura: Thoracotremata: Grapsoidea, Ocypodoidea. Vol. 21, 1–83. National Institute of Biological Resources Ministry of Environment.

[ece371712-bib-0048] Komai, T. 2011. “A New Species of the Varunid Crab Genus *Gopkittisak* (Crustacea: Decapoda: Brachyura: Grapsoidea) From the Ryukyu Islands.” Species Diversity 16, no. 3: 103–111. 10.12782/specdiv.16.103.

[ece371712-bib-0049] Komai, T. , R. O. Gotoh , T. Sado , and M. Miya . 2019. “Development of a New Set of PCR Primers for eDNA Metabarcoding Decapod Crustaceans.” Metabarcoding and Metagenomics 3: e33835. 10.3897/mbmg.3.33835.

[ece371712-bib-0050] Lasley, R. M., Jr. , A. Anker , and T. Naruse . 2024. “Two New Species and a New Record of Infaunal Crabs (Decapoda: Brachyura: Pinnotheridae and Varunidae) From Oman and Saudi Arabia.” Zootaxa 5476, no. 1: 207–229. 10.11646/zootaxa.5476.1.19. 39646449

[ece371712-bib-0051] Lee, S. H. , K. H. Lee , and H. S. Ko . 2010. “First Record of Holothurian Symbiotic Crab *Asthenognathus inaequipes* (Decapoda: Brachyura: Varunidae) From Korea.” Korean Journal of Systematic Zoology 26, no. 3: 337–339. 10.5635/KJSZ.2010.26.3.337.

[ece371712-bib-0052] Manning, R. B. , and L. B. Holthuis . 1981. “West African Brachyuran Crabs (Crustacea: Decapoda).” Smithsonian Contributions to Zoology 306: 1–379. 10.5479/si.00810282.306.

[ece371712-bib-0053] Mathews, L. M. , C. D. Schubart , J. E. Neigel , and D. L. Felder . 2002. “Genetic, Ecological, and Behavioural Divergence Between Two Sibling Snapping Shrimp Species (Crustacea: Decapoda: *Alpheus*).” Molecular Ecology 11, no. 8: 1427–1437. 10.1046/j.1365-294X.2002.01539. 12144663

[ece371712-bib-0054] McLay, C. L. , J. Kitaura , and K. Wada . 2010. “Behavioural and Molecular Evidence for the Systematic Position of *Macrophthalmus* (*Hemiplax*) *hirtipes* Hombron and Jacquinot, 1846, With Comments on Macrophthalmine Subgenera.” In Studies on Malacostraca: Lipke Bijdeley Holthuis Memorial Volume, vol. 14, 483–503. Crustaceana Monographs.

[ece371712-bib-0055] Miller, M. A. , W. Pfeiffer , and T. Schwartz . 2010. “Creating the CIPRES Science Gateway for Inference of Large Phylogenetic Trees.” In Proceedings of the Gateway Computing Environments Workshop (GCE), 1–8. CIPRES.

[ece371712-bib-0056] Monod, T. 1933. “Brachyura Maroccana. I. – Pinnotheridae, avec la description d'*Astenognathus atlanticus* nov. sp.” Bulletin de la Société des Sciences Naturelles du Maroc 13: 142–155.

[ece371712-bib-0057] Monod, T. 1956. Hippidea et Brachyura Ouest‐Africains. Vol. 45, 1–674. Mémoires de l'Institut Français d'Afrique Noire (IFAN).

[ece371712-bib-0058] Muñoz, I. 2024. Colección de Crustáceos Marinos (CRUST‐IEOCD). Version 1.31. Instituto Español de Oceanografía. Centro Oceanográfico de Cádiz (CSIC).

[ece371712-bib-0059] Naruse, T. , and P. F. Clark . 2009. “Establishment of a New Genus for *Asthenognathus gallardoi* Serène & soh, 1976 Within Gaeticinae Davie & N.K. Ng, 2007 (Crustacea: Decapoda: Brachyura: Varunidae).” Zootaxa 1987: 61–68.

[ece371712-bib-0060] Ng, N. K. 2006. The Systematics of the Crabs of the Family Varunidae (Brachyura, Decapoda). ScholarBank@NUS Repository.

[ece371712-bib-0061] Ng, P. K. L. , and P. J. F. Davie . 2024. “On the Identity of *Paranotonyx curtipes* Nobili, 1906 (Brachyura, Thoracotremata, Varunidae).” Crustaceana 97, no. 3–4: 289–305. 10.1163/15685403-bja10361.

[ece371712-bib-0062] Ng, P. K. L. , D. Guinot , and P. J. F. Davie . 2008. “Systema Brachyurorum: Part I. An Annotated Checklist of Extant Brachyuran Crabs of the World.” Raffles Bulletin of Zoology, Supplement 17: 1–286.

[ece371712-bib-0063] Ng, P. K. L. , and P.‐H. Ho . 2023. “The Sediment‐Dwelling Crabs of the *Tritodynamia hainanensis* Species‐Group (Crustacea: Decapoda: Brachyura: Macrophthalmidae), With Description of a New Species, *Tritodynamia wangi*, From Taiwan.” Journal of the National Taiwan Museum 76, no. 3/4: 199–213. 10.6532/JNTM.202312_76(3_4).11.

[ece371712-bib-0064] Ng, P. K. L. , and D. L. Rahayu . 2016. “On the Genera *Selwynia* Borradaile, 1903, and *Gandoa* Kammerer, 2006, With Descriptions of Two New Species From Papua New Guinea and French Polynesia (Crustacea: Decapoda: Brachyura: Aphanodactylidae).” Zootaxa 4092: 339–370. 27394459 10.11646/zootaxa.4092.3.2

[ece371712-bib-0065] Noël, P. , and F. Ziemski . 1933. “DORIS, 11/11/2020: *Asthenognathus atlanticus* Monod.” https://doris.ffessm.fr/Especes/Asthenognathus‐atlanticus‐Asthenognathe‐atlantique‐1869.

[ece371712-bib-0066] Noël, P. Y. , and J. M. Amouroux . 1977. “Sur la Presence D' *Asthenognathus atlanticus* Monod, 1932 (Crustacea, Brachyura) Dans la Region de Banyuls‐Sur‐Mer (Mediterranee).” Vie et Milieu, Ser. A 27, no. 1: 135–136.

[ece371712-bib-0067] Palacios‐Theil, E. , J. A. Cuesta , E. Campos , and D. L. Felder . 2009. “Molecular Genetic Re‐Examination of Subfamilies and Polyphyly in the Family Pinnotheridae (Crustacea: Decapoda).” In Decapod Crustacean Phylogenetics, edited by J. W. Martin , K. A. Crandall , and D. L. Felder , vol. 18, 457–474. CRC Press, Taylor & Francis Group.

[ece371712-bib-0068] Pezy, J.‐P. , and J.‐C. Dauvin . 2016. “Extension of the Geographical Distribution of the Crab *Asthenognathus atlanticus* Monod, 1932, in the Eastern English Channel Through Its Commensal Relationship With the Polychaete *Chaetopterus variopedatus* (Renier, 1804).” Marine Biodiversity 48: 987–993. 10.1007/s12526-016-0619-6.

[ece371712-bib-0069] Poore, G. C. B. , and S. T. Ahyong . 2023. Marine Decapod Crustacea: A Guide to Families and Genera of the World, 916. CSIRO Publishing. 10.1071/9781486311798.

[ece371712-bib-0070] Rambaut, A. 2012. FigTree, v.1.4.2. University of Edinburgh. http://tree.bio.ed.ac.uk/software/figtree/.

[ece371712-bib-0071] Rathbun, M. J. 1909. “New Crabs From the Gulf of Siam.” Proceedings of the Biological Society of Washington 22: 107–114.

[ece371712-bib-0072] Ronquist, F. , M. Teslenko , P. van der Mark , et al. 2012. “MrBayes 3.2: Efficient Bayesian Phylogenetic Inference and Model Choice Across a Large Model Space.” Systematic Biology 61: 539–542. 22357727 10.1093/sysbio/sys029 PMC3329765

[ece371712-bib-0073] Sakai, K. , M. Türkay , and S.‐L. Yang . 2006. “Revision of the *Helice*/ *Chasmagnathus* Complex (Crustacea: Decapoda: Brachyura).” Abhandlungen der Senckenbergischen Naturforschenden Gesellschaft 565: 1–77.

[ece371712-bib-0089] Schubart, C. D. , J. Reimer , and R. Diesel . 1998. “Morphological and Molecular Evidence for a New Endemic Freshwater Crab, *Sesarma ayatum* sp. n., (Grapsidae, Sesarminae) from Eastern Jamaica.” Zoologica Scripta 27, no. 4: 373–380. 10.1111/j.1463-6409.1998.tb00468.x.

[ece371712-bib-0074] Schubart, C. D. , C. Cannicci , M. Vannini , and S. Fratini . 2006. “Molecular Phylogeny of Grapsoid Crabs (Decapoda, Brachyura) and Allies Based on Two Mitochondrial Genes for a Proposal for Refraining From Current Superfamily Classification.” Journal of Zoological Systematics and Evolutionary Research 44, no. 3: 193–199. 10.1111/j.1439-0469.2006.00354.x.

[ece371712-bib-0075] Schubart, C. D. , J. A. Cuesta , R. Diesel , and D. L. Felder . 2000. “Molecular Phylogeny, Taxonomy, and Evolution of Nonmarine Lineages Within the American Grapsoid Crabs (Crustacea: Brachyura).” Molecular Phylogenetics and Evolution 15: 179–190. 10.1006/mpev.1999.0754. 10837150

[ece371712-bib-0077] Schubart, C. D. , J. I. González‐Gordillo , N. Reyns , H.‐C. Liu , and J. A. Cuesta . 2001. “Are Atlantic and Indo‐Pacific Populations of the Rafting Crab, *Plagusia depressa* , Distinct? New Evidence From Larval Morphology and mtDNA.” Raffles Bulletin of Zoology 49, no. 2: 301–310.

[ece371712-bib-0078] Schubart, C. D. , and M. G. J. Huber . 2006. “Genetic Comparisons of German Populations of the Stone Crayfish, *Austropotamobius torrentium* (Crustacea: Astacidae).” Bulletin Français de la Pêche et de la Pisciculture 380–381: 1019–1028. 10.1051/kmae:2006008.

[ece371712-bib-0079] Senckenberg . 2024. “Collection Crustacea SMF.” Occurrence dataset 10.15468/mc7ysi. Accessed via GBIF.org on 2024‐08‐03.

[ece371712-bib-0080] Spalding, M. D. , H. E. Fox , G. R. Allen , et al. 2007. “Marine Ecoregions of the World: A Bioregionalization of Coastal and Shelf Areas.” Bioscience 57, no. 7: 573–583. 10.1641/B570707.

[ece371712-bib-0081] Stamatakis, A. 2006. “RAxML‐VI‐HPC: Maximum Likelihood‐Based Phylogenetic Analyses With Thousands of Taxa and Mixed Models.” Bioinformatics 22: 2688–2690. 16928733 10.1093/bioinformatics/btl446

[ece371712-bib-0082] Števčić, Z. 2005. “Reklasifikacija Kratkorepih Rakova (Crustacea: Decapoda: Brachyura) [the Reclassification of Brachyuran Crabs (Crustacea: Decapoda: Brachyura)].” Natura Croatica 14, no. suppl. 1: 1–159.

[ece371712-bib-0083] Stimpson, W. 1858. “Prodromus Descriptionis Animalium Evertebratorum, Quae in Expeditione Ad Oceanum Pacificum Septentrionalem, a Republica Federata Missa, Cadwaladaro Ringgold et Johanne Rodgers Ducibus, Observavit et Descripsit. Pars V. Crustacea Ocypodoidea.” Proceedings of the Academy of Natural Sciences of Philadelphia 10: 93–110.

[ece371712-bib-0084] Tiedemann, M. , H. O. Fock , P. Brehmer , J. Döring , and C. Möllmann . 2017. “Does Upwelling Intensity Determine Larval Fish Habitats in Upwelling Ecosystems? The Case of Senegal and Mauritania.” Fisheries Oceanography 26: 655–667. 10.1111/fog.12224.

[ece371712-bib-0085] Tsang, C. T. , C. D. Schubart , K. H. Chu , P. K. Ng , and L. M. Tsang . 2022. “Molecular Phylogeny of Thoracotremata Crabs (Decapoda, Brachyura): Toward Adopting Monophyletic Superfamilies, Invasion History Into Terrestrial Habitats and Multiple Origins of Symbiosis.” Molecular Phylogenetics and Evolution 177: 107596. 35914646 10.1016/j.ympev.2022.107596

[ece371712-bib-0086] Wangensteen, O. S. , C. Palacín , M. Guardiola , and X. Turon . 2018. “DNA Metabarcoding of Littoral Hardbottom Communities: High Diversity and Database Gaps Revealed by Two Molecular Markers.” PeerJ 2018, no. 5: e4705. 10.7717/peerj.4705. PMC5937484 29740514

[ece371712-bib-0087] Wong, K. J. H. , L. S. R. Tao , and K. M. Y. Leung . 2021. “Subtidal Crabs of Hong Kong: Brachyura (Crustacea: Decapoda) From Benthic Trawl Surveys Conducted by the University of Hong Kong, 2012 to 2018.” Regional Studies in Marine Science 48: 102013. 10.1016/j.rsma.2021.102013.

[ece371712-bib-0088] Yang, S. L. , and B. P. Tang . 2008. “Description of the Male of *Asthenognathus hexagonum* Rathbun, 1909 (Decapoda, Pinnotheridae).” Crustaceana 81: 595–600. https://www.jstor.org/stable/20111422.

